# Brain imaging studies of emotional well-being: a scoping review

**DOI:** 10.3389/fpsyg.2023.1328523

**Published:** 2024-01-05

**Authors:** Caroline G. Richter, Celine Mylx Li, Adam Turnbull, Stephanie L. Haft, Deborah Schneider, Jie Luo, Denise Pinheiro Lima, Feng Vankee Lin, Richard J. Davidson, Fumiko Hoeft

**Affiliations:** ^1^Department of Psychology, University of Alabama at Birmingham, Birmingham, AL, United States; ^2^Department of Psychological Sciences, University of Connecticut, Storrs, CT, United States; ^3^Department of Psychology, University of California, Berkeley, Berkeley, CA, United States; ^4^Department of Brain and Cognitive Sciences, University of Rochester, Rochester, NY, United States; ^5^CogT Lab, Department of Psychiatry and Behavioral Sciences, Stanford University, Stanford, CA, United States; ^6^Intensive Care Pediatrician, Pediatric Intensive Care Unit, Hospital Moinhos de Vento, Porto Alegre, Brazil; ^7^Center for Healthy Minds, University of Wisconsin, Madison, WI, United States; ^8^Department of Psychology, University of Wisconsin, Madison, WI, United States; ^9^Waisman Laboratory for Brain Imaging and Behavior, University of Wisconsin, Madison, WI, United States; ^10^Department of Psychiatry, University of Wisconsin, Madison, WI, United States; ^11^Haskins Laboratories, New Haven, CT, United States; ^12^Brain Imaging Research Center (BIRC), University of Connecticut, Storrs, CT, United States; ^13^Department of Psychiatry and Behavioral Sciences, and Weill Institute for Neurosciences, University of California San Francisco, San Francisco, CA, United States; ^14^Department of Neuropsychiatry, Keio University School of Medicine, Shinanomachi Shinjuku Tokyo, Tokyo, Japan

**Keywords:** emotional well-being, neuroimaging, scoping review, positive affect, life satisfaction, sense of meaning, goal pursuit

## Abstract

This scoping review provides an overview of previous empirical studies that used brain imaging techniques to investigate the neural correlates of emotional well-being (EWB). We compiled evidence on this topic into one accessible and usable document as a foundation for future research into the relationship between EWB and the brain. PRISMA 2020 guidelines were followed. We located relevant articles by searching five electronic databases with 95 studies meeting our inclusion criteria. We explored EWB measures, brain imaging modalities, research designs, populations studied, and approaches that are currently in use to characterize and understand EWB across the literature. Of the key concepts related to EWB, the vast majority of studies investigated positive affect and life satisfaction, followed by sense of meaning, goal pursuit, and quality of life. The majority of studies used functional MRI, followed by EEG and event-related potential-based EEG to study the neural basis of EWB (predominantly experienced affect, affective perception, reward, and emotion regulation). It is notable that positive affect and life satisfaction have been studied significantly more often than the other three aspects of EWB (i.e., sense of meaning, goal pursuit, and quality of life). Our findings suggest that future studies should investigate EWB in more diverse samples, especially in children, individuals with clinical disorders, and individuals from various geographic locations. Future directions and theoretical implications are discussed, including the need for more longitudinal studies with ecologically valid measures that incorporate multi-level approaches allowing researchers to better investigate and evaluate the relationships among behavioral, environmental, and neural factors.

**Systematic review registration:**

https://osf.io/t9cf6/.

## 1 Introduction

According to the Centers for Disease Control and Prevention ([CDC], 2014), well-being generally refers to “judging life positively and feeling good,” yet there is no consensus around a single definition of well-being, and research indicates that well-being is a multifaceted construct. Many factors contribute to perceived well-being, including mental and physical health, social relationships, and quality of life (Diener, 1984; Organisation for Economic Co-operation and Development, 2020). Out of all the aspects of well-being, the focus of this review is on emotional well-being (EWB). EWB is not synonymous with the absence of negative states such as depressed or anxious thoughts or feelings, but instead comprises an important independent domain of positive functioning (Feller et al., 2018; Organisation for Economic Co-operation and Development, 2020).

Definitions of EWB differ widely across the literature, and the diversity of methods and measures used to study EWB reflect a lack of consensus of this concept. In an effort to build consensus around the construct and advance research on EWB, NIH funded six EWB High Priority Research Networks (RFA-AT-20-003) from across the country in early 2021, whose main goals included the development of a working definition of EWB (National Institute of Health [NIH], 2018). The work of this NIH-funded consortium led to the following working definition for EWB: “EWB is a multi-dimensional composite that encompasses how positive an individual feels generally and about life overall. It includes both experiential features (emotional quality of momentary and everyday experiences) and reflective features (judgments about life satisfaction, sense of meaning, and ability to pursue goals that can include and extend beyond the self). These features occur in the context of culture, life circumstances, resources, and life course.” (Park et al., 2023a, p. 16). Importantly, the working definition of EWB includes both evaluative (e.g., life satisfaction, sense of meaning) and experienced (e.g., positive mood, day-to-day feelings of happiness) aspects of emotion, as well as hedonic (i.e., positive affect and life satisfaction) and eudaimonic aspects (i.e., sense of meaning in life and goal pursuit) of positive affect. As emphasized by Park et al. (2023a,b) this definition of EWB is still evolving, and aspects of the definition can be mapped onto the well-established constructs of subjective well-being (Diener, 1984) and psychological well-being (Ryff, 1989). Furthermore, Park et al. (2023b) subsequent commentary clarifies that although this definition emphasizes the positive aspects of EWB, it is designed to encompass varying degrees of positive experience and affect. Moreover, it is designed to capture the importance of negative emotional experiences in shaping the emotional quality of quotidian and moment-to-moment experiences.

Previous studies have demonstrated that elevated EWB is associated with decreased mortality risk and increased physical and psychological health (Feller et al., 2018; Trudel-Fitzgerald et al., 2021). Research has consistently linked higher levels of happiness to an increased likelihood of maintaining healthy lifestyles over extended periods, with one longitudinal study reporting evidence of this relationship over 12 years (Trudel-Fitzgerald et al., 2019). Furthermore, elevated EWB is correlated with a lower risk of chronic illnesses such as cardiovascular disease and diabetes. It also appears to contribute to healthier aging and increased longevity (Boehm and Kubzansky, 2012; Pressman et al., 2019; Steptoe, 2019). Meta-analyzes indicate that individuals with higher EWB, compared to those with lower levels, have a 17% reduced risk of mortality (Cohen et al., 2016), a 14% decrease in all-cause mortality risk (Rozanski et al., 2019), and a notable 25% reduction in mortality risk among older adults (Zhang and Han, 2016).

**Figure 1:**
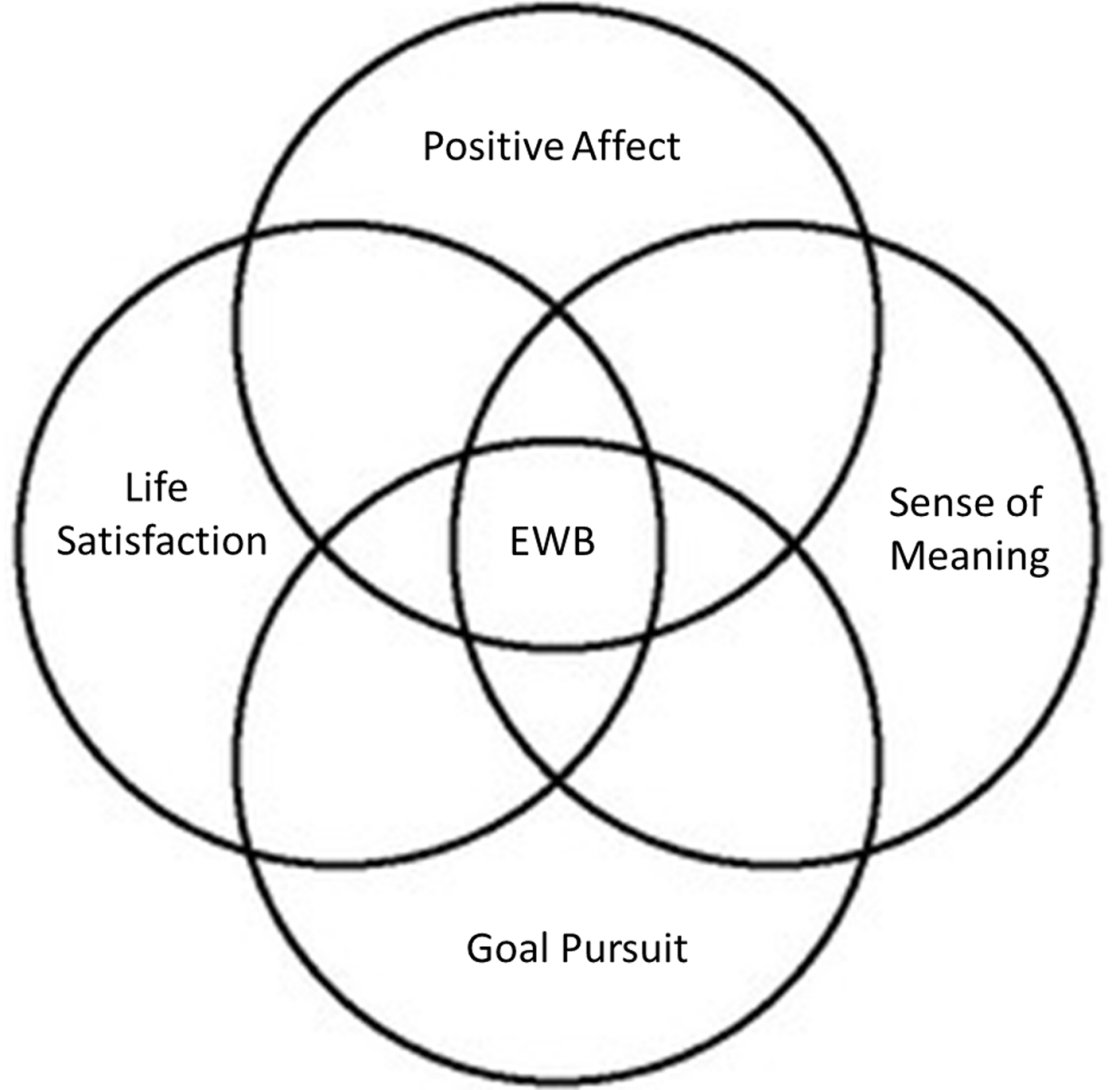
Graphic illustration of key emotional well-being constructs.

In the present scoping systematic review, we seek to contribute to the scientific understanding of EWB by organizing existing knowledge in the field into a coherent and accessible framework and by making neuroimaging recommendations for future research into the neural processes related to EWB. Our scoping review is organized following this working definition of EWB (see Figure 1).

### 1.1 Overview of EWB neuroimaging research

EWB broadly relates to the subjective experience of the *goodness* of one’s life and, thus, has traditionally been evaluated using self-report instruments. Neuroimaging research, by contrast, has contributed to our understanding of the brain-based mechanisms underlying EWB and may help researchers to identify new avenues for interventions to increase EWB. It may also someday provide a measure of EWB in populations with cognitive or communication differences that can make self-report measures unreliable, such as those with severe or profound intellectual disabilities (Flynn et al., 2017). Non-invasive brain imaging is widely used to understand neural mechanisms in humans and is particularly relevant for the study of EWB, given the limits of examining EWB in animal models (but see Weiss et al., 2011; Ben-Shaanan et al., 2016). Neuroimaging has enhanced our understanding of emotional processing and paved the way for innovative treatments for emotional challenges. As an example, studies of the brain regions and networks most affected by depression have informed the development of brain stimulation techniques that demonstrate promising outcomes in treating depressive symptoms (Cash et al., 2021). An improved understanding of the neural correlates of EWB may allow for the development of similar interventions to improve EWB in groups at-risk for low EWB and the broader population and identification of potential biomarkers for those interventions.

Research indicates that EWB is linked to variations in network connectivity, both within and between networks, as well as the dynamic interactions therein (Shi et al., 2018). A number of large-scale brain networks, such as the default mode, salience, and frontoparietal networks, have been associated with EWB (Lindquist et al., 2012; Kragel and LaBar, 2016; Riedel et al., 2018). These networks have also been linked with self-reflection (van der Meer et al., 2010), interoception (Kleckner et al., 2017), theory of mind (Schurz et al., 2014), emotion regulation (Morawetz et al., 2020), and cognitive control (Cocchi et al., 2013).

Both regional and network-level brain variations appear to correlate to aspects of EWB. However, there is limited agreement amongst study findings and a dearth of well-defined theories that add explanatory value and predictive power to these results. Moreover, consistent with trends in neuroimaging more broadly (Siddiqi et al., 2022), few studies provide causal insight into the neural foundation of EWB. This is largely due to a lack of temporal clarity (i.e., whether brain changes precede behavioral ones), experimental manipulation (i.e., if altering the brain influences behavior), and convergence amongst study findings (i.e., whether research consistently highlights a specific brain region or network).

The variety and complexity of literature investigating the neural basis of EWB might stem from the methodological variability inherent in neuroimaging studies (Carp, 2012), including differences in MRI techniques (e.g., structural MRI, resting-state fMRI, task-based fMRI), fMRI tasks, and preprocessing strategies. Additionally, the low reproducibility of this research (Poldrack et al., 2020) complicates drawing definitive conclusions from individual studies. A systematic investigation of neuroimaging research on EWB is necessary to lay the groundwork for future studies and to evaluate the readiness of specific sub-domains of EWB and its neural correlates for eventual meta-analysis. Such meta-analyzes should ideally encompass studies with consistent methodologies (Müller et al., 2018). While systematic reviews are known to have biases, such as publication bias, inclusion of low-quality articles, and lack of homogeneity on the data summarized (e.g., Murad et al., 2018; Uttley et al., 2023), they nevertheless provide clear advantages over individual studies or narrative reviews, by offering a more impartial assessment of the existing evidence (e.g., Finckh and Tramèr, 2008; Haddaway et al., 2020).

Motivated by the aforementioned identified need and formal efforts to better operationalize EWB, we undertook a systematic review of neuroimaging studies that include behavioral measures of constructs related to EWB. Additionally, we sought to capture various definitions and conceptualizations of EWB and how they have evolved over time in relation to neuroimaging, as well as identify gaps in the extant literature. It’s important to emphasize that our review does not seek to identify or establish the neural correlates of EWB directly; the diversity in design, imaging modality, and behavioral measures across included studies renders comparison and conclusion difficult, if not impossible. Our aim, rather, is to present a current overview of the literature, paving the way for more focused future research on the neural underpinnings of EWB.

### 1.2 Previous reviews of EWB neuroimaging research

Our team conducted a systematic search of previously published reviews—including chapters, narrative reviews, systematic reviews, and meta-analyzes—that explored concepts related to EWB and incorporated any form of brain imaging. Out of the 17 reviews we identified: Four examined the neural correlates of well-being, encompassing concepts including overall well-being, positive affect, happiness, and psychological well-being (Keverne, 2004; Huppert, 2009; King, 2019; Alexander et al., 2021); four focused on positive and negative emotions (Critchley, 2003; Murphy et al., 2003; Vytal and Hamann, 2010; Machado and Cantilino, 2017); three examined well-being in specific scenarios or amongst specific populations, such as the relation between aesthetic emotion and psychological well-being and aging (Kryla-Lighthall and Mather, 2009; St. Jacques et al., 2013; Mastandrea et al., 2019); three specifically targeted happiness (Subramaniam and Vinogradov, 2013; Suardi et al., 2016; Tanzer and Weyandt, 2020); two evaluated neural processes linked to mindfulness practices by using mindfulness as their EWB-related measure probed with neuroimaging (Marchand, 2014; Kaur and Singh, 2015); and one focused on the neural foundation of anomalies in emotional stimuli processing in individuals with mood disorders (Leppänen, 2006).

With respect to previously published systematic reviews of constructs related to EWB (e.g., Keverne, 2004; Huppert, 2009; Subramaniam and Vinogradov, 2013; Suardi, 2016; King, 2019; Tanzer and Weyandt, 2020; Alexander et al., 2021), our current scoping review distinguishes itself in several important ways: (1) We focused specifically on EWB, aligning our search with the working definition proposed by Park et al. (2023a). (2) Our inclusion criteria required that the brain imaging studies also include at least one subjective measure of constructs related to EWB identified by the scoping review of reviews conducted by Koslouski et al. (2022) that compiled a list of EWB measures extracted from previous reviews. (3) Instead of limiting our focus to a specific imaging method, we included a broad spectrum of imaging modalities. (4) We sought to identify broad trends in existing research rather than focus narrowly on the neural correlates of EWB, a task better suited for future meta-analyzes within brain imaging modalities.

### 1.3 The present review

The objective for the present study was to undertake a scoping review of past empirical studies employing brain imaging techniques and self-report measures to explore the neural and behavioral underpinnings of EWB. Identifying, describing, and synthesizing prior works on this subject is of considerable value to the scientific community as it offers insights into the progression of EWB research, refines our understanding of the construct, and paves the way for subsequent inquiries in this domain. Specifically, this review investigates imaging modalities and measures used to capture and comprehend EWB. Furthermore, it describes and analyzes trends in extant research, encompassing research design, target population, imaging methodologies, and findings, as well as how these have evolved over time.

## 2 Methods

### 2.1 Search strategy

**Figure 2:**
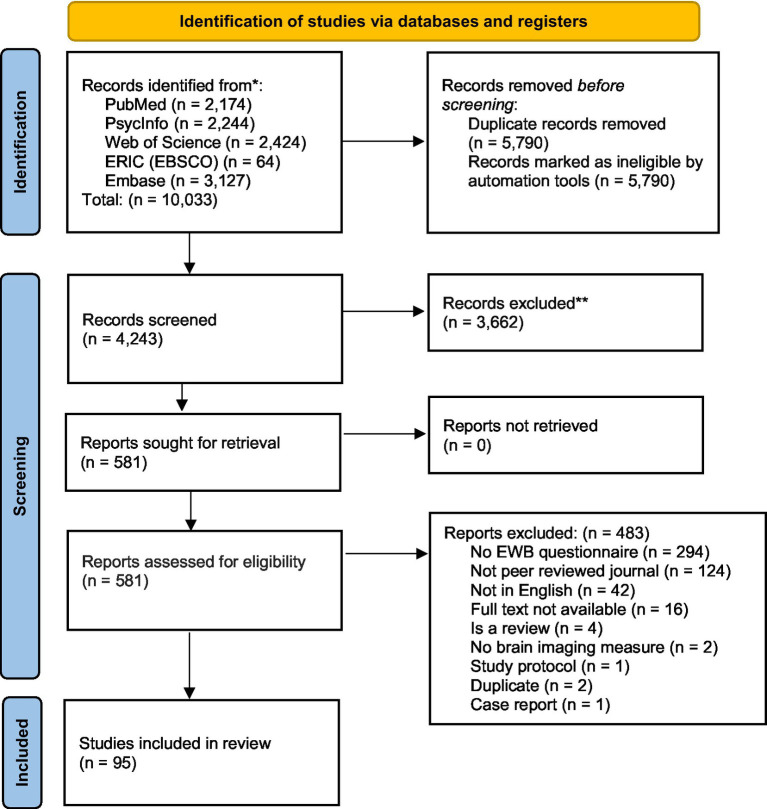
PRISMA 2020 flow diagram for the systematic search on brain imaging studies of emotional well-being. Page et al. (2021) for more information, visit: http://www.prisma-statement.org/.

This is a scoping review of the literature on brain imaging studies including measures of EWB. We followed the PRISMA 2020 guidelines (Page et al., 2021) in conducting and reporting our search and preregistered this study on Open Science Framework on July 15, 2021 (doi: 10.17605/OSF.IO/T9CF6). We located relevant articles by searching five electronic databases: PubMed, PsycInfo, Web of Science, ERIC (EBSCO), Embase, with the latest search conducted on July 09, 2021. Complete and detailed searches per database can be found in Supplemental materials. To obtain the maximum number of articles, we elected not to restrict our search by date. Articles originating in any nation were included, but searches were limited to articles published in peer reviewed journals and in English. Following each database’s guidelines, we entered keywords that combined terms related to EWB and terms related to brain imaging modalities. Articles that included at least one search term from each Group (A: EWB and its components, B: brain imaging modalities, and C: brain or neuro-related terms) were captured (see Supplementary Table S1) for screening and eventual review. Based on this search, we identified a total of 4,243 articles after duplicates were removed (see Figure 2 for PRISMA 2020 flow diagram).

### 2.2 Inclusion and exclusion criteria

We reviewed the articles for inclusion based on the following inclusion criteria: (1) The use of at least one brain imaging modality; (2) At least one measure of EWB or its components (see list in Supplementary Table S2 andrationale below); (3) Articles published in peer reviewed journals; (4) Studies published in English. We excluded articles based on the following criteria: (1) Book chapters, reviews, case studies, qualitative studies, meta-analysis, systematic reviews; (2) Unrelated articles, duplicates, unavailable full texts, or abstract-only papers; (3) Articles published on Google Scholar only, dissertations, theses, conference papers, opinion papers; and (4) Animal research. We did not impose restrictions on publication date, methodological rigor, characteristics of participants, or age of participants included in the study. As the current study is a scoping review, study quality was not investigated (Munn et al., 2018).

### 2.3 Search procedures

Search procedures comprised three separate stages: (1) Title and abstract screening, (2) Full-text screening, and (3) Data extraction. At each stage, relevant procedures were conducted independently by at least two trained research assistants using *Covidence* (systematic review software; Veritas Health Innovation, 2021). Discrepancies at each stage were resolved by a third rater (authors of this current article) also using Covidence. We followed the procedures outlined by Polanin et al. (2019), including the creation of a checklist file with specific screening questions that guided raters throughout the screening process (see Supplemental materials).

To pass to the full-text screening phase, each study must have included at least one of the 135 EWB measures outlined in Supplementary Table S2 (a comprehensive list of these measures, with citations, can be found in Supplementary Table S2). These measures originated from a scoping review of reviews that gathered questionnaires designed to capture individual experiences of EWB (Koslouski et al., 2022). Consistent with our operational definition of EWB, instruments focusing solely on depression, anxiety, or other negative emotions were omitted from their assembled list. Our scoping review was conducted concurrently with that of Koslouski et al. (2022), employing analogous search terms, with the intention of yielding a cohesive set of outcomes to further enrich the existing literature.

### 2.4 EWB constructs

As previously noted, our scoping review follows the theoretical framework proposed by Park et al. (2023a). Accordingly, we classified the included studies into five core EWB domains as conceptualized by Park et al. (2023a): (1) positive affect, (2) life satisfaction, (3) goal pursuit, (4) quality of life, and (5) sense of meaning. These classifications were based on the EWB measures employed by the studies included in this review.

### 2.5 Brain imaging modalities

Studies were classified into three main categories based on their design and purpose. Studies were characterized as task-based functional imaging studies if they examined the function of the brain using a task-based paradigm. Task-based imaging methods included fMRI, EEG, ERP, PET, SPECT, TMS, and tDCS. For the task-based functional imaging studies, we provided a brief description of the task or paradigm used in the study, along with the main EWB domain investigated in the study (e.g., experienced affect, affective perception, reward, and emotion regulation). Studies were characterized as resting-state if functional images were acquired under resting-state conditions, using methods such as fMRI, EEG, PET, MRS, rTMS, and TBS. Finally, those studies examining structural properties of the brain such as grey and white matter properties, were categorized as structural MRI studies and included methods such as MRI and resting DTI. For studies in all three categories, main regions of interest (ROI) were documented, and main brain outcome measures were also reported.

## 3 Results

### 3.1 Study and sample characteristics

**Figure 3:**
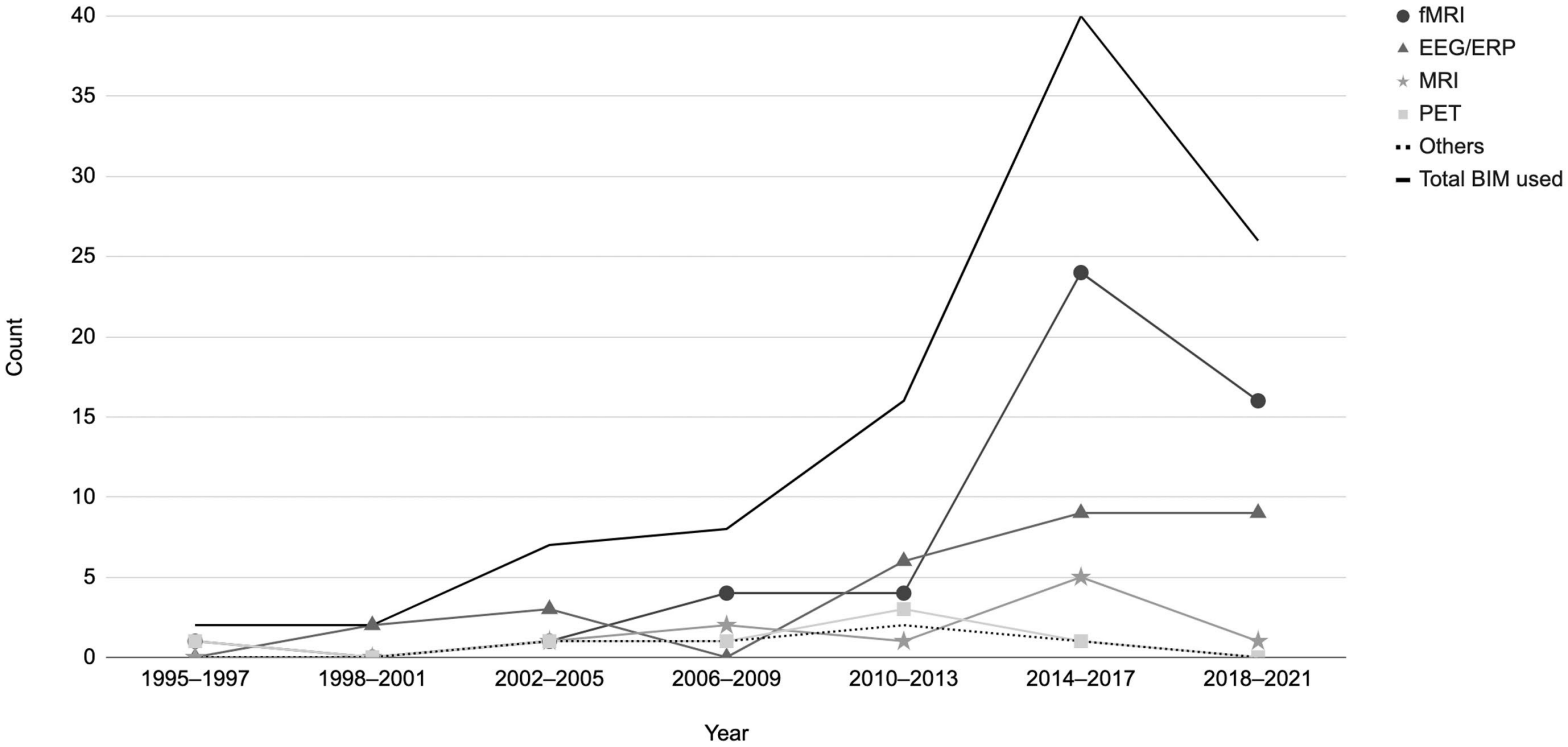
Distribution of papers by year based on the brain imaging modalities utilized (fMRI, EEG/ERP, MRI, PET). Others = TMS, rTMS/TBS, tDCS, SPECT and MRS. A total of 101 brain imaging modalities used across the 95 studies included in the current review.

The distribution of papers by year can be found in Figure 3. There was a clear increase in the number of articles published after 2010, with a peak in 2015 (n = 16), then a slight decline. Of the 95 articles in this review, 69 were published between 2013 and 2021. This trend corresponds with the advancements in brain imaging techniques and their growing use in EWB studies.

Table 1 presents the sample characteristics of the 95 studies included in this review and Supplementary Table S3 presents the studies’ aims and main findings. A minority (*n* = 14) was conducted with clinical populations. This includes one study with a subclinical group and another with an at-risk group. The specific disorders investigated were: post-traumatic stress disorder (*n* = 3), schizophrenia (*n* = 1), major depressive disorder or depression (*n* = 6), social anxiety disorder (*n* = 1), chronic lower back pain (*n* = 2), and primary insomnia (*n* = 1). The large majority of the studies (*n* = 81) involved samples reported to be non-clinical, not at risk, or healthy.

**Table 1:** Characteristics of studies included for analysis (*N* = 95).

First Author (Year)	Country	Sample (*N*)	Study design	Clinical Sample Characteristics	Age Groups	Sex (% female)	Age mean (SD)	Age (min – max)	Race %	EWB Questionnaire(s)
Alessandri et al. (2015)	Italy	51	Cross-sectional	—	Young adults, Adults	100	24.1 (3.7)	20–34	—	LOT-R; SWLS
Arjmand et al. (2017)	Australia	18	Cross-sectional	—	Adolescents, Adults	66.7	22.22 (5)	18–38	—	PANAS
Baeken et al. (2008)	Belgium	47	non-RCT	—	Adults	100	25.4 (—)	—	—	PANAS
Costa et al. (2019)	Italy	17	Cross-sectional	—	Young adults, Adults	58.8	25.06 (5.1)	20–40	—	PANAS; SWLS
Cunningham and Kirkland (2014)	United States	42	Cross-sectional	—	—	50	—	—	—	PANAS; SHS
Davidson et al. (2003)	United States	41	RCT	—	Adults	70.1	36 (—)	23–56	White 95.1%; Asian 4.9%	PANAS
Day et al. (2019)	Australia	69	Cross-sectional	Chronic lower back pain	Young adults	52	51 (14.4)	18 –	White 88%; Asian 4%; Other 8%	SHS
Dennison et al. (2015)	Australia	89	Cohort	—	Adolescents	48.3	12.6 (0.5)	—	—	PANAS
Dolcos et al. (2021)	United States	19	non-RCT	PTSD	—	5	30.9 (7.9)	—	White 79%; Black 11%; Asian 5%; Other 5%	PANAS
Flores et al. (2018)	United States	34	Cross-sectional	—	Adolescents	65	16.3 (1.5)	14–18	White 79%; Black 15%; Other or multiracial 6%	PANAS-C
Fonzo et al. (2017)	United States	66	non-RCT	PTSD	Young adults, Adults	65.2	36.7 (—)	18–60	—	WHOQOL-BREF
Geethanjali et al. (2019)	India	16	Cross-sectional	—	—	—	—	—	—	PANAS
George et al. (1995)	United States	11	Cross-sectional	—	—	100	33.3 (12.3)	—	—	PANAS
Gondoh et al. (2009)	Japan	30	non-RCT	—	—	36.6	21.1 (—)	—	—	SHS
Habel et al. (2004)	Germany	52	Case–control study	Schizophrenia	Young adults, Adults	0	33.3 (—)	18–45	—	PANAS
Hagemann et al. (1999)	Germany	36	Cross-sectional	—	—	66.6	23.5 (—)	—	—	PANAS
Hall and Petruzzello (1999)	United States	41	Cross-sectional	—	Adults, Older adults	63.4	68.7 (5.8)	60–85	—	PANAS; SWLS
Hasler et al. (2012)	United States	27	Case–control study	Primary Insomnia	Young adults, Adults	55.6	37.8 (—)	22–51	—	PANAS
He et al. (2019)	China	68	Case–control study	Major Depressive Disorder	—	58.8	35.5 (—)	—	—	PANAS
Heller et al. (2013a)	United States	35	non-RCT	Major Depressive Disorder	Young adults	54.34	31.7 (—)	19–60	—	PANAS
Heller et al. (2013b)	United States	64	Cross-sectional	—	Adults, Older adults	62.5	58.2 (11.4)	38–79	White 65.6%; Black 31.2%; Other 3.1%	PANAS; RPWB
Hiyoshi-Taniguchi et al. (2015)	Japan	12	Cross-sectional	—	—	83.3	21.9 (0.3)	—	—	PANAS
Hofer et al. (2007)	Austria	38	Cross-sectional	—	Young adults, Adults	50	33 (—)	20–48	—	PANAS
Isbel et al. (2019)	Australia	67	RCT	—	—	62.8	71.0 (—)	—	—	PANAS
Jung et al. (2002)	United States	27	Cross-sectional	—	Young adults, Adults	40.7	24.9 (5.8)	18–37	—	PANAS
Katsumi et al. (2021)	Japan	70	Cross-sectional	—	Young adults	100	21.7 (1.8)	—	—	SHS
Kawamichi et al. (2016)	Japan	113	Case–control study	—	Young adults	43.5	21.4 (—)	—	—	SHS
Killgore and Yurgelun-Todd (2007)	United States	13	Cross-sectional	—	Young adults, Adults	100	23.5 (2.1)	21–28	—	PANAS
Koepp et al. (2009)	United Kingdom	25	Cross-sectional	—	Adults	28	—	30–52	—	PANAS
Kohn et al. (2014)	Germany	54	Cross-sectional	—	—	51.9	29.9 (8.2)	—	—	PANAS
Kong et al. (2015a)	China	294	Cross-sectional	—	—	53.8	21.6 (1)	—	—	PANAS; SWLS
Kong et al. (2015b)	China	276	Cross-sectional	—	Young adults	53.9	21.6 (1.0)	18–25	—	SWLS
Kong et al. (2015c)	China	286	Cross-sectional	—	Young adults	54.1	21.6 (1)	—	—	RPWB
Kong et al. (2016)	China	290	Cross-sectional	—	—	54.1	21.6 (1.0)	—	—	PANAS; RPWB
Kong et al. (2018)	China	100	Cross-sectional	—	Young adults	58	20.9 (2)	18–26	—	PANAS; SWLS
Kujawa et al. (2015)	United States	381	Cohort	—	Children	45.1	3.6 (0.3)	—	White 94.8%; Black 2.9%; Asian 2.4%; Hispanic or Latino/a; 7.6%	AFARS
Kwon et al. (2021)	South Korea	83	Cross-sectional	—	—	50.6	22.9 (—)	—	—	SWLS
Kyeong et al. (2020)	South Korea	48	Cross-sectional	—	—	50	22.7 (—)	—	—	SWLS
Larson et al. (2010)	United States	45	Cross-sectional	—	Young adults, Adults	51.1	21.3 (2.4)	18–29	—	LOT-R; PANAS; SWLS
Lewis et al. (2014)	United Kingdom	70	Cross-sectional	—	—	60	24.6 (3.8)	—	—	RPWB
Li et al. (2011)	China	27	Cross-sectional	—	Young adults, Adults	74	22.4 (2.5)	19–28	—	PANAS
Li et al. (2016)	China	23	Cross-sectional	—	—	34.8	23.5 (—)	—	—	PANAS
Lichev et al. (2015)	Germany	46	Cross-sectional	—	—	50	23.5 (2.7)	—	—	PANAS
Liu et al. (2015)	Taiwan	66	Cross-sectional	—	—	53.2	21.6 (—)	—	—	PANAS
Lorenzetti et al. (2009)	Australia	89	Case–control study	Major Depressive Disorder	—	68.5	33.8 (—)	—	—	PANAS
Love et al. (2012)	United States	55	Cross-sectional	—	—	58.2	26.5 (—)	—	—	PANAS; RPWB
Luo et al. (2014)	China	50	Case–control study	—	—	72	20.3 (—)	—	—	PANAS; SHS
Luo et al. (2017)	China	138	Cross-sectional	—	—	62.3	21.1 (1.7)	—	—	PANAS; RPWB
Ma et al. (2015)	China	46	non-RCT	—	Young adults	0	19 (—)	18–23	—	PANAS
Martikainen et al. (2015)	United States	32	Case–control	Chronic non-neuropathic back pain	—	43.7	35 (—)	—	—	PANAS
Martin-Soelch et al. (2020)	Switzerland	32	Case–control study	Relatives of Major Depressive Disorder	Young adults, Adults	75	24.8 (—)	20–37	—	PANAS
Mathiak et al. (2013)	Germany	13	Cross-sectional	—	Young adults	0	—	18–26	—	PANAS
Matsunaga et al. (2016)	Japan	132	Cross-sectional	—	Young adults, Adults	54.6	21.7 (—)	18–34	—	SHS
McPhee et al. (2021)	Australia	28	RCT	—	Adults, Older adults	57.1	66.9 (—)	57–78	—	CASP-19
Memarian et al. (2017)	United States	113	non-RCT	—	—	46.9	21 (—)	—	—	SWLS
Mennella et al. (2017)	Italy	32	RCT	—	—	100	23.1 (1.2)	—	—	PANAS
Miskowiak et al. (2008)	United Kingdom	24	RCT	—	—	58.3	23.5 (—)	—	—	PANAS
Morelli et al. (2018)	United States	46	Cross-sectional	—	—	50	19.3 (1.2)	—	White 41%; Black 11%; Asian15%; Hispanic or Latino/a 13%; Pacific Islander 4%; Mixed 13%; Other 3%	PANAS; SWLS
Mrazek et al. (2016)	United States	31	non-RCT	—	—	48.4	21.5 (2.2)	—	—	PANAS; SWLS
Nardo et al. (2011)	Sweden	30	Case–control study	PTSD	—	19.9	42.4 (—)	—	—	WHO
Oetken et al. (2017)	Germany	21	Cross-sectional	—	Young adults, Adults	52.3	26.5 (5.5)	21–42	—	PANAS
Park et al. (2017)	Switzerland	50	RCT	—		78	25.6 (0.7)	—	—	SHS
Plitnick et al. (2010)	United States	22	non-RCT	—	Young adults, Adults	59	—	19–27	—	PANAS
Puccetti et al. (2021)	United States	52	Cross-sectional	—	Adults, Older adults	67	57.7 (10.5)	39–76	White 69%; Black 29%; Native American or Alaskan Native 2%	RPWB
Radsepehr et al. (2019)	Iran	33	Cross-sectional	—	Young adults, Adults	48	—	19–29	—	PANAS
Rohr et al. (2015)	Germany	43	Cross-sectional	—		53.4	25.8 (2.4)	21–30	—	PANAS
Sailer et al. (2016)	Sweden	25	Cross-sectional	—	Young adults, Adults	60	23 (3.9)	19–38	—	PANAS
Sanchez et al. (2015)	Brazil	22	Cross-sectional	—	Young adults, Adults	45.5	26.3 (4.5)	19–37	—	PANAS
Sanger et al. (2018)	United Kingdom	40	non-RCT	—	Adolescents	—	16.8 (0.6)	—	—	WHO
Sato et al. (2005)	Japan	18	Cross-sectional	—	Young adults, Adults	44.4	25.7 (3.4)	20–31	—	PANAS
Sato et al. (2015)	Japan	51	Cross-sectional	—	—	51	22.5 (4.5)	—	—	SHS
Sato et al. (2019)	Japan	51	Cross-sectional	—	—	50.1	22.5 (4.5)	—	—	SHS
Schmitt et al. (2019)	Germany	21	non-RCT	—	—	0	27.1 (4.1)	—	—	PANAS
Schneider et al. (1997)	Germany	12	Cross-sectional	—	Adults	41.7	29.7 (4.3)	25–36	—	PANAS
Schöne et al. (2018)	Germany	34	RCT	—	—	76.5	21.2 (—)	—	—	PANAS
Shi et al. (2016)	China	50	Cross-sectional	—	—	48	21 (1.5)	—	—	PANAS
Shi et al. (2019)	China	212	Cross-sectional	—	Young adults, Adults	54.2	22.4 (1.5)	19–27	—	PANAS
Singleton et al. (2014)	United States	14	non-RCT	—	Adults	64.3	37.9 (4.3)	29–44	White 78.6%; Black 7.1%; Asian 7.1%; Multiracial 7.1%	RPWB
Spironelli et al. (2020)	Italy	62	Case–control study	Major Depressive Disorder	—	69.3	53.2 (—)	—	—	PANAS
Suslow et al. (2015)	Germany	34	Cross-sectional	—	—	100	24.7 (3.3)	—	—	PANAS
Talmon et al. (2021)	United States	269	Case–control study	Social Anxiety Disorder	—	—	—	—	—	SWLS
Urry et al. (2004)	United States	84	Cross-sectional	—	Adults	48.8	58.5 (0.81)	57–60	—	PANAS; RPWB; SWLS
van Reekum et al. (2007)	United States	29	Cross-sectional	—	Adults	62.1	63.5 (—)	61–65	—	RPWB
Vanderhasselt et al. (2013)	Brazil	25	non-RCT	—	—	68	22.1 (3.8)	—	—	PANAS
Velikova and Nordtug (2017)	—	20	non-RCT	—	Young adults	50	37.9 (—)	22–51	—	SWLS
Volkow et al. (2011)	United States	47	Cross-sectional	—	—	51.1	31.5 (—)	—	—	MPQ-WB
Wang et al. (2020)	China	31	Cross-sectional	—	—	45.2	20.2 (0.3)	—	—	PANAS
Waytz et al. (2015)	United States	84	Cross-sectional	—	—	51.2	25.3 (9.9)	—	—	RPWB; MLQ; SWLS
Woods et al. (2020)	United States	96	Cross-sectional	—	Adolescents	30.2	16.3 (—)	14–18	—	PANAS-C
Xu et al. (2018)	China	48	RCT	—	—	56.25	22 (1.8)	—	—	PANAS; SWLS
Yu and Li (2012)	China	36	Cross-sectional	—	—	50	21.6 (—)	—	—	MUNSH
Zhang et al. (2014)	China	16	Cross-sectional	—	—	0	27 (4)	—	—	PANAS
Zhang et al. (2015)	China	25	Cross-sectional	—	Young adults	56	18.6 (—)	18–24	—	PANAS
Zhang et al. (2017)	China	50	Case–control study	Depression	—	70.01	32.5 (—)	—	—	PANAS
Zubieta et al. (2003)	United States	14	Cross-sectional	—	—	100	36 (9)	—	—	PANAS

LOT-R, Life Orientation Test-Revised; SWLS, Satisfaction with Life Scale; PANAS, Positive and Negative Affect Schedule; SHS, Subjective Happiness Scale; PANAS-C, Positive and Negative Affect Schedule for Children; WHO, World Health Organization Well-Being Index; WHOQOL-BREF, World Health Organization Quality of Life – BREF; RPWB, Ryff’s Scales of Psychological Well-Being; AFARS, Affect and Arousal Scale; CASP-19, Quality of Life scale; MPQ-WB, Multidimensional Personality Questionnaire Well-being Scale; MLQ, Meaning in Life Questionnaire; MUNSH, Scale of Happiness of the Memorial University of Newfoundland.

With respect to developmental stages, we classified studies based on the mean age reported in the articles. Seven studies did not provide this information. Of the remaining 88 studies, the preponderance (*n* = 82) focused on adults (age ≥ 18). Only four of these included older adults in their sample (age > 65; Hall and Petruzzello, 1999; Heller et al., 2013b; McPhee et al., 2021; Puccetti et al., 2021). Five studies included adolescents (age 12–18) in their sample (Dennison et al., 2015; Arjmand et al., 2017; Flores et al., 2018; Sanger et al., 2018; Woods et al., 2020), and one study, by Kujawa et al. (2015), included children aged 12 or younger.

Only nine of the included articles reported the racial or ethnic composition of their samples, and all but one (Day et al., 2019; conducted in Australia) of these were conducted in the United States (Davidson et al., 2003; Heller et al., 2013b; Singleton et al., 2014; Kujawa et al., 2015; Flores et al., 2018; Morelli et al., 2018; Dolcos et al., 2021; Puccetti et al., 2021). Amongst the nine studies reporting racial or ethnic demographic data, the majority of the participants were White (ranging from 65.6 to 95.1%). Black/African American participants ranged from 2.9 to 31.3% of the total sample, whilst Asian participants varied between 2.4 and 15% of the total sample. Three studies (Singleton et al., 2014; Flores et al., 2018; Morelli et al., 2018) included a multiracial category. Only two studies (Kujawa et al., 2015; Morelli et al., 2018) mentioned the inclusion of Hispanic or Latino/a participants (see Table 1 for details).

With respect to socio-economic status (SES), 16 studies explicitly reported information on education, and only two reported income of participants. Thirty-six studies reported university student participants, suggesting their samples had some level of college education; however, factors explicitly related to SES were not reported.

### 3.2 Journals

Studies included in this review were published in a variety of peer reviewed journals (*n* = 52) varying widely in impact factor (see Supplementary Table S4). Specifically, nine of the studies were published in *NeuroImage* and another nine in *Social Cognitive and Affective Neuroscience*, followed by 6 in *Frontiers in Human Neuroscience* and 4 in the *American Journal of Psychiatry*. To evaluate the impact of journals, we used the five-year impact factor (2017–2021) from *Journal Citation Reports (JCR) - Clarivate*. This impact factor is calculated by dividing the total citations in 2021 from articles published in 2016 to 2020 by the total number citable articles in 2016–2020. The JCR impact factor of the journals ranged from 2.826 (i.e., *Social Neuroscience*) to 19.59 (i.e., *American Journal of Psychiatry*) for the 18 journals that have two or more studies included in this current review.

### 3.3 Countries

Most of the articles included in the current review were conducted in the United States (*n* = 29) and China (*n* = 19), followed by Germany (*n* = 11). It is noteworthy that the studies published in North America, Europe, and Australia/Oceania are shown to be overrepresented in proportion to the world population share; on the other hand, articles published in Asia, Africa, and South America appear to be underrepresented to the world population share (see Supplementary Figure S1).

### 3.4 EWB measures used and EWB constructs investigated

Supplementary Table S5 reports EWB measures and constructs across included studies. Thirteen of the 135 EWB measures identified by Koslouski et al. (2022), were employed in the 95 studies, with a reported total of 115 instances of use. This indicates multiple EWB measures were used in some studies. The Positive and Negative Affect Schedule (PANAS; Watson et al., 1988) was most commonly used at 68.4% of the studies (excluding 2.1% using the children’s version). This was followed by the Satisfaction with Life Scale (SWLS; Diener, 1984) at 17.9%. The Subjective Happiness Scale (SHS; Lyubomirsky and Lepper, 1999) and Ryff’s Psychological Well-being Scale (PWB; Ryff, 1989) had similar frequencies of use, at 10.5 and 11.6%, respectively. Another nine measures were employed in 12 studies.

Of the studies examined, all 95 incorporated at least one measure designed to evaluate the constructs of positive affect, mood, or emotion. All but one examined the construct of life satisfaction. This overlap in constructs stems from the fact that many EWB measures are designed to probe both areas. For example, PANAS is a widely-used instrument measuring both life satisfaction and positive affect. Far fewer studies measured more evaluative aspects of EWB, including sense of meaning (14 studies), goal pursuit (13 studies), and quality of life (2 studies).

### 3.5 Study design

As reported in Table 1, a preponderance of studies were cross-sectional (*n* = 59), followed by 22 intervention studies, 12 case–control studies, and only two cohort studies. A description of the intervention studies can be found in Table 2. Of these,13 used a quasi-experimental (non-randomized) design, and eight constituted randomized controlled trials (RCTs). These intervention studies can be further classified into six distinct categories: (1) psychological interventions (*n* = 6), (2) mindfulness-based interventions (*n* = 6), (3) pharmacological interventions (*n* = 4), (4) physical exercise interventions (*n* = 3), (5) non-invasive brain stimulation techniques (*n* = 2), and (6) light-exposure based interventions (*n* = 1). It is important to note that few of these studies aimed at drawing causal inferences about the neural basis of EWB: Instead, most sought to identify and describe behavioral and neural correlates of their respective interventions. For example, Dolcos et al. (2021) examined changes in EWB measures and brain measures following cognitive-emotional training but did not establish a causal link between neural and behavioral changes.

**Table 2:** Characteristics of intervention Studies (*N* = 22).

	Non-RCT (*n* = 14)	RCT (*n* = 8)
Psychological intervention (*n* = 6)	Cognitive-emotional regulation training program (Dolcos et al., 2021) Prolonged exposure treatment (Fonzo et al., 2017) 12-week lasting self-guided positive imagery training (Velikova and Nordtug, 2017)	Cognitive training + *Bacopa monnieri* (McPhee et al., 2021) Neurofeedback training (Mennella et al., 2017) Positive psychological intervention (Xu et al., 2018)
Mindfulness (*n* = 6)	School-Based Mindfulness Training (Sanger et al., 2018) Mindfulness-based intervention (Singleton et al., 2014) Intensive multifaceted intervention: physical exercise + formal mindfulness practice + lecture or discussion (Mrazek et al., 2016)	Mindfulness Meditation (Davidson et al., 2003) Mindfulness training intervention (Isbel et al., 2019) Mindful breath awareness meditation (Schöne et al., 2018)
Pharmacological interventions (*n* = 4)	Treatment with either fluoxetine or venlafaxine (Heller et al., 2013b) SSRI (30 mg citalopram) (Ma et al., 2015)	Commitment to spend money on others *vs* self (Park et al., 2017) Injection of Erythropoietin (Miskowiak et al., 2008)
Physical exercise (*n* = 3)	Aerobic exercise training (Gondoh et al., 2009) Low-and high-intensity exercise (Schmitt et al., 2019) Intensive multifaceted intervention: physical exercise + formal mindfulness practice + lecture or discussion (Mrazek et al., 2016)	
Non-invasive brain stimulations (*n* = 2)	Active HF-rTMS (Baeken et al., 2008) Sham-controlled tDCS (Vanderhasselt et al., 2013)	
Light exposure (*n* = 1)	Light exposure (Plitnick et al., 2010)	

RCT, randomized controlled trial; SSRI, Selective serotonin reuptake inhibitor.

### 3.6 Brain imaging modalities

Among the 95 studies included in this review, neuroimaging modalities were used 101 times, indicating that some studies used more than one modality. Functional magnetic resonance imaging (fMRI) was the predominant modality, used in 50 studies. Continuous electroencephalogram (EEG) and event-related potentials (ERPs) were employed in 24 and five studies, respectively. A limited number of studies (*n* = 9) used structural MRI. Both magnetic resonance spectroscopy (MRS) and diffusion-weighted imaging (DWI) were used in a single study. Positron emission tomography (PET) imaging was employed in 7 of the studies, whereas single-photon emission computerized tomography (SPECT) was used in only single study. This distribution underscores a pronounced pattern of functional over structural investigations.

Complementary to these imaging methods, certain studies incorporated neuromodulatory techniques. Notably, transcranial magnetic stimulation (TMS), transcranial direct current stimulation (tDCS), and repetitive TMS/theta-burst stimulation (TBS) each appeared in a singular study (refer to Tables 3–5 for details).

**Table 3:** Task-based functional imaging studies (*N* = 57).

First Author (Year)	BIM(s)	Task Paradigm/description	Domain	ROIs	Brain outcome measure
Arjmand et al. (2017)	EEG	Music Mood Induction/ Stimuli	Experienced affect	Mid-frontal (F3/4, FC3/4)	Alpha asymmetry
Costa et al. (2019)	fMRI	Imagination Tasks: Sentence Stimuli	Experienced affect	Whole-brain	BOLD activity
Cunningham and Kirkland (2014)	fMRI	IAPS (Lang et al., 1998)	Experienced affect	Amygdala	BOLD activity
Davidson et al. (2003)	EEG	Emotion induction: writing task	Experienced affect	Anterior (F3/4, FC7/8, T3/4, C3/4)	Alpha asymmetry
Flores et al. (2018)	fMRI	Monetary-reward fMRI task, Social Reward Task (Davey et al., 2010)	Reward	“Social” brain regions (from Neurosynth)	BOLD activity
Fonzo et al. (2017)	TMS, fMRI	Emotion Reactivity Task (Etkin et al., 2004), Emotional Conflict Task (Etkin et al., 2006), Reappraisal Task (Minkel et al., 2012), & Gender Conflict Task (Egner et al., 2008)	Emotion regulation	Whole-brain, Amygdala, aIns, FPC	BOLD activity, seed-based functional connectivity (task-related)
Geethanjali et al. (2019)	EEG	Mood Induction: Music Stimuli	Experienced affect	Whole-brain	Alpha, beta, and theta band energy
George et al. (1995)	PET	Mood induction	Experienced affect	Whole-brain	Cerebral blood flow
Habel et al. (2004)	fMRI	Mood Induction (Schneider et al., 1997)	Experienced affect	Amygdala, Thalamus, Precuneus, ACC, OFC, STC, dlPFC	BOLD activity
Heller et al. (2013a)	fMRI	IAPS (Lang et al., 1998)	Affective perception	NAcc, PFC	BOLD activity, seed-based functional connectivity (task-related)
Heller et al. (2013b)	fMRI	IAPS (Lang et al., 1998)	Emotion regulation	Whole-brain	BOLD activity
Hiyoshi-Taniguchi et al. (2015)	EEG	Emotional Judgment	Affective perception	Whole-brain	Alpha, beta, and theta relative power
Hofer et al. (2007)	fMRI	Mood Induction (Schneider et al., 1997)	Experienced affect	Whole-brain	BOLD activity
Killgore and Yurgelun-Todd (2007)	fMRI	Stimulation tasks: viewing images of high calorie and low calorie foods	Affective perception	Occipital lobe	BOLD activity
Koepp et al. (2009)	PET	Mood induction	Experienced affect	Whole-brain	DPN receptor binding (opioid)
Kohn et al. (2014)	fMRI	Mood Induction (Schneider et al., 1997)	Experienced affect	Whole-brain	BOLD activity
Kujawa et al. (2015)	EEG,ERP	Monetary Reward Task	Reward	Frontal midline (Fz, FCz, Cz)	Feedback negativity
Kyeong et al. (2020)	fMRI	Self-evaluation tasks: Self-Criticism Task, Self-Respect Task	Experienced affect	PCC, vmPFC, NAcc	Seed-based functional connectivity
Larson et al. (2010)	ERP	Response inhibition tests: Eriksen Flanker Task	Performance monitoring	Frontal midline (FCz, Cz)	Error-related negativity, post-error positivity
Li et al. (2011)	fMRI	Decision-making: Loss Decision Task, Visual Perceptual Decision Task	Reward	Whole-brain, Amygdala	BOLD activity
Li et al. (2016)	fMRI	rtfMRI-nf training paradigm	Emotion regulation	Whole-brain, Ins, Amygdala, ACC, dmPFC	BOLD activity
Lichev et al. (2015)	fMRI	Facial emotion recognition	Affective perception	Whole-brain, Amygdala	BOLD activity
Liu et al. (2015)	EEG	Monetary reward: Dynamic Reward Task	Reward	Midline (Fz, Cz, Pz), parietal (P7/8), frontal midline (Fz, FCz, Cz)	N170, early emotional positivity, feedback-related negativity
Love et al. (2012)	PET	Pain task	Pain	Whole-brain	D2/D3 receptor binding (dopamine)
Ma et al. (2015)	fMRI	Chinese Facial Affective Picture System, Repetition-Detection Task	Affective perception	Whole-brain, Amygdala	BOLD activity
Martikainen et al. (2015)	PET	Pain task	Pain	Whole-brain	D2/D3 receptor distribution volume ratio (dopamine), K ratio (11C[raclopride] tracer transport)
Martin-Soelch et al. (2020)	fMRI	Monetary reward: Fribourg Reward Task	Reward	Whole-brain, Cd, Put, Pallidym, NAcc	BOLD activity
Mathiak et al. (2013)	fMRI	Playing a violent video game	Experienced affect	Whole-brain	BOLD activity
Matsunaga et al. (2016)	fMRI	Life Event Imagination Task	Experienced affect	Whole-brain	BOLD activity, gray matter density
Memarian et al. (2017)	fMRI	Affect Labeling	Affective perception	Whole-brain	BOLD activity
Mennella et al. (2017)	EEG	Real-time neurofeedback task: emotion regulation	Emotion regulation	Frontal (F3/4)	Alpha asymmetry, alpha power
Miskowiak et al. (2008)	fMRI	Gender discrimination task	Affective perception	Whole-brain, Amygdala	BOLD activity
Morelli et al. (2018)	fMRI	Card-Guessing Task, Monetary Incentive Delay task	Reward	Whole-brain, vStr, vmPFC, mPFC	BOLD activity
Nardo et al. (2011)	SPECT	Traumatic memory task	Experienced affect	Whole-brain	cerebral blood flow
Oetken et al. (2017)	fMRI	Mood Induction: Music stimuli; Self-Evaluation Task	Experienced affect	Whole-brain	BOLD activity
Park et al. (2017)	fMRI	Decision-Making Task	Reward	Whole-brain, vStr, OFC	BOLD activity, seed-based functional connectivity (task-related), edgewise connectivity (task-related)
Plitnick et al. (2010)	EEG	Light Exposure	Experienced affect	Whole-brain	Alpha, alpha-theta, beta, theta, and gamma relative power
Puccetti et al. (2021)	fMRI	IAPS (Lang et al., 1998)	Affective perception	Amygdala	BOLD activity
Radsepehr et al. (2019)	EEG	Mood induction: Video clips	Experienced affect	Whole-brain	absolute alpha power
Sailer et al. (2016)	fMRI	External stimuli: Robot arm stroking/touching	Affective perception	Whole-brain, pIns	BOLD activity, seed-based connectivity
Sanchez et al. (2015)	fMRI	Discrimination/Judgment task	Affective perception	Whole-brain, Amygdala	BOLD activity
Sanger et al. (2018)	ERP	Facial/emotional recognition: Emotional oddball	Affective perception	Centroparietal (CP2/4, P2/4)	P3b amplitude
Sato et al. (2005)	EEG	Monetary reward: Two-choice decision-making task	Reward	Midline (Fz, Cz, Pz)	Feedback negativity, P300
Schmitt et al. (2019)	fMRI	Face-Processing fMRI paradigm, Radboud Faces Database of Facial Affect series (Langner, et al., 2010)	Affective perception	Whole-brain	BOLD activity
Schneider et al. (1997)	fMRI	Mood induction task, cognitive task	Experienced affect	Amygdala, cingulum, STC, ITC, frontal white matter, SMC, Ins	BOLD activity
Schöne et al. (2018)	EEG	Multiple object tracking (MOT) paradigms	Visual attention	Whole-brain	Steady-state visually evoked potential
Shi et al. (2016)	fMRI	Self-evaluation: Personality Trait Adjective Pool	Sense of self	Whole-brain	BOLD activity
Spironelli et al. (2020)	EEG	Phonological task, Semantic task	Linguistic/semantic	Frontoparietal (F3/4/7/8/9/10, FC3/4, P3/4/7/8, TP7/8, O1/2)	Normalized alpha amplitude, normalized high-beta amplitude
Suslow et al. (2015)	fMRI	Bodily Expressive Action Stimulus Test	Affective perception	Amygdala, Striatum, Thalamus	BOLD activity
Talmon et al. (2021)	fMRI	Self-evaluation: self-judgment task	Sense of self	mPFC, PCC	BOLD activity
van Reekum et al. (2007)	fMRI	IAPS (Lang et al., 1998)	Affective perception	Amygdala, PFC	BOLD activity
Vanderhasselt et al. (2013)	EEG, ERP, tDCS	Cued Emotional Control Task (CECT)	Affective perception	dlPFC (tDCS), whole-brain	N450
Woods et al. (2020)	fMRI	Best Friend fMRI task	Social perception	Whole-brain	BOLD activity
Yu and Li (2012)	EEG	Emotion-priming Diagram, IAPS (Lang et al., 1998)	Affective perception	Midline (Fz, Cz, Pz)	late positive potential
Zhang et al. (2017)	ERP	Cyberball	Social exclusion	Frontal midline (Fz, FCz, Cz)	P3 amplitude
Zhang et al. (2015)	fMRI	Chinese Affective Picture System	Affective perception	Emotion regions (56 ROIs)	Network and nodal graph theory indices
Zubieta et al. (2003)	MRI, PET	Mood induction	Experienced affect	Whole-brain	μ-opioid receptor availability

BIM, Brain imaging modalities; (a/p)Ins, (Anterior/Posterior) Insula; FPC, frontopolar cortex; ACC, Anterior Cingulate Cortex; OFC, Orbitofrontal Cortex; (S/I)TC, (Superior/Inferior) Temporal Cortex; dl/dm/vm/mPFC, (Dorsolateral/Dorsomedial/Ventromedial/Medial) Prefrontal Cortex; PCC, Posterior Cingulate Cortex; NAcc, Nucleus Accumbens; Cd, Caudate; Put, Putamen; Pallidum; (v)Str, (Ventral) Striatum; SMC, Sensorimotor Cortex.

**Table 4:** Resting-state studies (*N* = 30).

First Author (Year)	BIM	ROIs	Brain outcome measure
Alessandri et al. (2015)	EEG	Whole-brain	Alpha asymmetry
Baeken et al. (2008)	rTMS or TBS	dlPFC	n/a
Day et al. (2019)	EEG	Mid-frontal (F3/F4)	Alpha asymmetry
Dolcos et al. (2021)	fMRI	Amygdala, dlPFC, vlPFC, mPFC	Seed-based functional connectivity
Hagemann et al. (1999)	EEG	Whole-brain	Alpha power density, alpha asymmetry
Hall and Petruzzello (1999)	EEG	Frontal regions (F3/4)	Alpha power density, alpha asymmetry
Hasler et al. (2012)	MRI, PET	Striatum, mPFC	Glucose metabolism
He et al. (2019)	fMRI	vlPFC, sgACC, Hippocampus, Amygdala, Cd, Put, SFG, MFG, IFG, OFC, Rec, Olf, Ins, PHG, cingulate, Pallidym, Thalamus	Edgewise functional connectivity
Isbel et al. (2019)	EEG	Frontal regions (Fp1/2, F3/4, F7/8)	Alpha asymmetry
Jung et al. (2002)	MRS	Frontal white matter, occipitoparietal white matter	N-acetylaspartate, creatine, and choline
Katsumi et al. (2021)	fMRI	Whole-brain	Seed-based functional connectivity
Kong et al. (2018)	fMRI	Whole-brain	fALFF
Kong et al. (2016)	fMRI	Whole-brain	ReHo
Kong et al. (2015a)	fMRI	Whole-brain	fALFF
Kong et al. (2015b)	fMRI	ACC, OFC, vmPFC, Ins, Thalamus, Hippocampus, Amygdala	ReHo
Kong et al. (2015c)	fMRI	Whole-brain	fALFF
Kwon et al. (2021)	fMRI	NAcc, OFC, sgACC, Ins	Seed-based functional connectivity
Luo et al. (2017)	fMRI	DMN	Within-network functional connectivity
Luo et al. (2014)	fMRI	Whole-brain	ReHo
Mrazek et al. (2016)	fMRI	Whole-brain, PCC	Seed-based functional connectivity, degree centrality
Rohr et al. (2015)	fMRI	Amygdala	Seed-based functional connectivity
Sato et al. (2019)	fMRI	Whole-brain, Amygdala	fALFF, seed-based functional connectivity
Shi et al. (2019)	fMRI	Ins, rACC, dACC, dlPFC, OFC	Seed-based functional connectivity
Urry et al. (2004)	EEG	Whole-brain	Alpha asymmetry
Velikova and Nordtug (2017)	EEG	Whole-brain	Current source density
Volkow et al. (2011)	PET	Whole-brain	Glucose metabolism
Wang et al. (2020)	EEG	PCC, amPFC	Power spectrum, EEG functional connectivity (power envelope correlation)
Waytz et al. (2015)	fMRI	DMN	Within-network functional connectivity
Xu et al. (2018)	EEG	Frontal regions (F3/4/7/8, FC3/4/5/6)	Alpha asymmetry
Zhang et al. (2014)	EEG	Whole-brain	Alpha, beta, and theta relative power, theta/beta ratio

BIM, Brain imaging modalities; (dl/vl//vm/m/am)PFC, (Dorsolateral/Ventrolateral/Ventromedial/Medial/Anterior Medial) Prefrontal Cortex; (a)Ins, (Anterior) Insula; FPC, Frontopolar Cortex; (sg/r/d)ACC, (Subgenual/Rostral/Dorsal) Anterior Cingulate Cortex; Hipp, Hippocampus; Cd, Caudate; Put, Putamen; (S/M/I)FG, (Superior/Middle/Inferior) Frontal Gyrus; OFC, Orbitofrontal Cortex; Rec, Rectus; Olf, Olfactory areas; PHG, Parahippocampal gyrus; Pallidym; NAcc, Nucleus Accumbens; DMN, Default Mode Network; PCC, Posterior Cingulate Cortex.

**Table 5:** Structural MRI studies (*N* = 8).

First Author (Year)	BIM	ROIs	Brain outcome measure
Dennison et al. (2015)	MRI	Total brain volume, Amygdala, Cd, Put, NAcc, Pallidym, Hippocampus	Total MRI volume
Gondoh et al. (2009)	MRI	Whole-brain	Gray matter volume
Kawamichi et al. (2016)	MRI	Striatum	Gray matter density
Lewis et al. (2014)	MRI	Whole-brain	Gray matter volume
Lorenzetti et al. (2009)	MRI	Pituitary gland	Total MRI volume
McPhee et al. (2021)	DTI	Whole-brain	White matter mean diffusivity/fractional anisotrophy, gray matter dispersion/neurite density
Sato et al. (2015)	MRI	ACC, Precuneus, Amygdala	Gray matter volume
Singleton et al. (2014)	MRI	Brainstem, cerebellum, PCC, TPJ	Gray matter concentration

BIM, Brain imaging modalities; Cd, Caudate; Put, Putamen; NAcc, Nucleus Accumbens; Pallidum; DMN, Default Mode Network; ACC, Anterior Cingulate Cortex; PCC, Posterior Cingulate Cortex; TPJ, Temporoparietal Junction.

**Figure 4:**
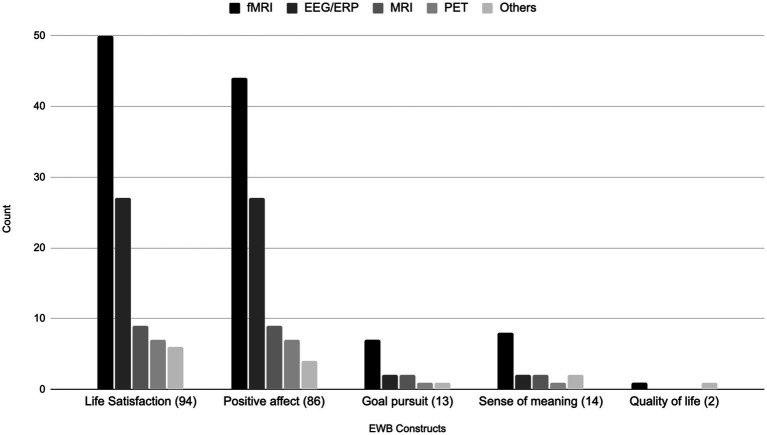
Bar chart of EWB constructs being investigated by brain imaging modalities. Others = TMS, rTMS/TBS, tDCS, SPECT and MRS.

Figure 4 details the use of various brain imaging modalities across five distinct EWB constructs. Amongst the 94 studies examining the construct of life satisfaction, fMRI emerged as the predominant modality, used in 50 studies. This was followed by EEG/ERP in 27 studies, MRI in nine studies, PET in seven studies, and other modalities in six studies. Of the 86 studies examining positive affect, fMRI was again used in a preponderance of studies (44 studies). EEG/ERP was used in 27 studies, MRI in nine studies, PET in seven studies, and other techniques were used in four studies. Of those examining goal pursuit, fMRI was employed in seven of 13 studies, and EEG/ERP and MRI were used in two studies each. PET and other techniques were each used in a single study. Amongst the 14 studies evaluating sense of meaning, fMRI was the modality of choice in eight. EEG/ERP and MRI were each employed in two studies, with PET used in one study, and other modalities in two studies. Collectively, these findings underscore a consistent preference for the use of fMRI across EWB constructs, highlighting its widespread use in the field.

### 3.7 Task-based functional imaging studies

Table 3 depicts the task-based functional imaging studies (*n* = 57). Among the 57 studies measuring brain activity during task performance, the most commonly used (*n* = 10) instrument was the International Affective Picture System (IAPS; Lang et al., 1998). Various mood induction tasks were employed in 18 studies (e.g., Schneider et al., 1997), and a range of other paradigms were also used, with most used only in one or two studies.

We classified study paradigms into domains corresponding to the cognitive or emotional processes they were designed to evaluate. These domains comprised: experienced affect, affective perception, emotion regulation, reward, linguistic/semantic processing, pain induction, performance monitoring, sense of self, social exclusion, social perception, and visual attention. As an example, paradigms designed to explore brain activity during distinct affective states, such as positive/negative moods or self-judgment, were categorized under the domain of *experienced affect*. Paradigms that required participants to modulate their emotional responses were classified as *emotion regulation* tasks. By contrast, those assessing brain activity in response to emotionally-valenced stimuli were classed as *affective perception*. Paradigms designed to probe affective perception included both passive viewing of stimuli (e.g., IAPS), as well as active tasks that asked participants to judge, recognize, label, and discriminate emotionally-valenced stimuli. Notably, the domains of experienced affect and affective perception predominated in our dataset, comprising 19 and 17 of the 57 task-based studies, respectively. These were followed by studies focused on emotion regulation (*n* = 4). This emphasis on the evaluation of neuroactivity underpinning emotion processing aligns with the EWB used measures in these studies, specifically those examining experiential facets of EWB, such as positive affect.

The next most commonly studied domain was reward, with eight studies measuring brain function during processes related to reward. Two studies measured brain activity during pain induction, and two during paradigms designed to explore sense of self. Several studies used paradigms aimed at understanding cognitive processes, including one performance monitoring task, one visual attention task, and one study using linguistic/semantic tasks. Finally, two studies used paradigms designed to measure brain activity during social processing, one using a social perception task (video clips of *best friends* vs. strangers) and one using a social exclusion task (Cyberball game, in which participants are excluded from a virtual game).

With respect to brain regions examined, 21 of task-based functional imaging studies used exploratory whole-brain approaches (looking for any relationships to either connectivity or activity across every brain region) with no *a priori* defined ROI. After taking this exploratory whole brain approach, most of these studies (*n* = 12) identified the regions that were most active during the chosen task, and then within those specific regions (ROI) investigated the relations with a particular EWB measure (see Table 3 for details). In studies with *a priori* ROIs, a number of EEG studies targeted frontal/mid-frontal electrodes as a means of calculating frontal asymmetry, a measure commonly linked to positive affect. In general, EEG studies used various approaches to quantify neural activity, including calculating energy/power within different frequency bands (mostly alpha, beta, and theta), as well as ERPs such as the feedback negativity response, N170, and P3. Across fMRI studies, a range of target regions were explored across both the cortex and subcortex. Frontal regions (e.g., dorsolateral prefrontal cortex, orbitofrontal cortex, anterior cingulate cortex) were more commonly targeted than others, although several studies examined the posterior cingulate cortex as a ROI. The insula and amygdala, two regions commonly linked to emotional processing, were also well represented. Other subcortical regions, including the striatum and thalamus, were also targeted in multiple studies. fMRI studies predominantly looked at brain activity measured using BOLD signal response, although several studies also looked at functional connectivity during task performance.

### 3.8 Resting-state studies

Table 4 presents those studies that used resting state methodologies to examine brain function, accounting for 30 studies. Of these, fMRI was used in 16 studies. EEG was employed in 10 studies, and PET in two, while rTMS or TBS and MRS were each used in a single study. Of note, almost half of these studies (*n* = 15) explored connectivity or activity across the entire brain, indicating an absence of *a priori* hypotheses focused on specific brain regions or networks. As with the task-based studies, several of the EEG studies targeted frontal/mid-frontal electrodes to calculate measures of alpha asymmetry. The fMRI resting state studies that did define ROIs *a priori* targeted a range of regions across all four cortical lobes, as well as the subcortex. Studies used a range of resting state analytical approaches, including seed-based functional connectivity, fractional amplitude of low-frequency fluctuations (fALFF), and regional homogeneity (ReHo) to measure brain function at rest. In line with the EEG studies, areas the frontal lobe were identified as ROIs more often than those in the occipital, parietal, or temporal lobes, with several studies targeting the orbitofrontal cortex, medial prefrontal cortex, dorsolateral prefrontal cortex, and anterior cingulate cortex (as was found for the task-based fMRI studies). Similar to task-based studies, the insula and amygdala were also commonly identified as ROIs. Two studies also used the default mode network as a network-of-interest, the only network to be targeted in this way.

### 3.9 Structural MRI studies

Table 5 includes studies that used structural MRI approaches (*n* = 8). Of these studies, one used DTI while the remainder used MRI. Three studies used whole brain approaches, while the others defined ROI *a priori*. In contrast with the task-based and resting state studies, the majority of regions targeted by structural MRI studies were subcortical regions, including the amygdala, striatum, and hippocampus, and one study targeting the pituitary gland. All studies (except the DTI study) in this category assessed gray matter structure, measuring total MRI volume, gray matter volume, gray matter density, and/or gray matter concentration. The single DTI study measured white matter mean diffusivity/fractional anisotropy and gray matter dispersion/neurite density.

## 4 Discussion and future directions

We conducted a scoping review of neuroimaging studies designed to explore and evaluate the relationship between EWB and the brain. By compiling, organizing, and assessing included articles, we sought to establish a foundation for future modality-specific meta-analyzes and provide insight into the current state of the evidence, as well as identify gaps in the literature and highlight directions for future research.

With respect to overall trends, few studies evaluated the neural correlates of EWB in populations other than White healthy young adults; as such, there is a notable gap in the research involving children, older adults, clinical populations, and more diverse, underrepresented groups and populations. It’s likewise evident that the neural basis of experienced affect, especially positive mood, has been better researched than the evaluative components of EWB. The majority of these studies used exploratory (whole-brain) imaging techniques, and among those with pre-determined ROIs, there was little consistency among targeted regions. This suggests a lack of consensus around the neural correlates of aspects of EWB. However, the existence of whole-brain maps of EWB associations suggests that future meta-analyzes, particularly leveraging novel cross-modality analyzes such as standard permutation of subject images (Albajes-Eizagirre et al., 2019), may be able to better summarize this research and provide greater clarity on which brain regions and networks are most strongly implicated in EWB to guide future research and intervention development.

While trait associations can be summarized—for example, those derived from resting state and structural MRI—a significant challenge arises due to the considerable variability in tasks employed to probe EWB. Aggregating data from varied tasks in meta-analyzes is rare due to the complexities associated with interpreting analogous activation patterns within diverse contexts. We must reiterate that our inclusive search criteria, encompassing diverse studies that included any form of neuroimaging and a wide variety of EWB measures, resulted in the inclusion of a large number of studies whose primary aims did not include the establishment of causal links between EWB and brain functioning. This diversity, coupled with a dearth of causal design, complicates summarization and renders generalization impossible. Nevertheless, we contend that our review offers valuable insights into the current state of the EWB neuroimaging literature, potentially informing subsequent focused research. Specifically, the findings from this review might guide the development of a taxonomy of tasks used in EWB research, categorizing them based on the aspects of EWB they measure and the brain regions they activate. In the remainder of the Discussion, we discuss the main findings of this study, limitations, and directions for future research.

### 4.1 EWB measures

The majority of included studies used PANAS; although previous research has demonstrated that this scale is a valid and reliable measure of certain aspects of EWB (e.g., Pavot et al., 1991; Crawford and Henry, 2004), the manner in which the instrument is used and interpreted can vary widely. For example, the PANAS questionnaire can be used as a tool to measure momentary affect (e.g., *Indicate to what extent you feel this way right now, that is, at the present moment.*) or trait affect (e.g., *Indicate to what extent you generally feel this way, that is how you feel on average*.). Yet, the majority of studies included in our sample did not describe how PANAS was used in their protocol (i.e., whether it was used as a measure of momentary affect or trait affect). In addition, several studies reported using the PANAS to evaluate changes in affect during tasks (e.g., emotion regulation and affect perception tasks), which suggests that the instrument was used to measure affect in response to task-specific stimuli, rather than to evaluate momentary or trait affect in daily life. Future studies should be more transparent while reporting on the use of questionnaires.

In light of PANAS’s widespread adoption in EWB assessment, it’s important to acknowledge its usefulness in efficiently measuring positive and negative affect. However, a potential weakness of the PANAS is its emphasis on high-arousal positive affect dimensions (e.g., alertness, excitation), to the exclusion of low-arousal positive emotions like calmness (*cf.* Pressman et al., 2019). The utility of the PANAS is tempered by its lack of scope, specifically its limitation in capturing a diverse range of discrete positive emotions. Future research should address this limitation by selecting or designing instruments designed to evaluate a broader range of EWB constructs in greater granularity. In this context, the circumplex model of affect presents a potential alternative (Russell, 1980).

The majority of the studies included in this review were task-based functional imaging studies. Of these studies, one third measured brain activity during an experienced affect task and 28 measured brain activity during affective perception (e.g., viewing affective images). Notably, a limited number of studies endeavored to discern associations between brain activity and responses on EWB questionnaires, potentially shedding light on the influence of trait EWB on neural functioning in specific contexts. Overall, the literature appears to exhibit a paucity of evidence concerning neural underpinnings of evaluative EWB as a trait.

While certain resting state and structural studies—which reflect trait aspects of brain function and structure— did examine the relationships between brain activity and evaluative EWB questionnaire outcomes (e.g., Urry et al., 2004; Kong et al., 2015a,b,c; Kwon et al., 2021), others analyzed these measures separately without seeking to establish correlational or causal links (e.g., Mrazek et al., 2016; Dolcos et al., 2021). In some cases, brain function was evaluated in relation to trait measures of experienced affect over extended periods, as observed in studies addressing the trait neural markers of subjective well-being (e.g., Luo et al., 2014; Sato et al., 2015, 2019; Katsumi et al., 2021).

### 4.2 Brain imaging modalities

Our findings indicate substantial variability not only in the use of instruments (e.g., the PANAS), but also in the use of stimuli across studies. For example, while the IAPS was used in six of the included studies, its application varied: Some studies used IAPS as a tool for mood induction (e.g., asking participants to describe images with the intent of affecting change in their mood, as measured by the PANAS; Cunningham and Kirkland, 2014), while others (e.g., van Reekum et al., 2007; Heller et al., 2013b) used IAPS to stimulate emotion regulation (e.g., suppression) or promote affective perception. As such, it is difficult to generalize findings, even amongst studies using the same stimuli.

This lack of uniformity in study design and use of measures and stimuli hampers our capacity to establish an integrated knowledge base on EWB. Given the multifaceted nature of EWB, which encompasses elements such as positive affect, life satisfaction, sense of meaning, and goal pursuit, a number of measures would likely be required to measure the construct holistically. Future research, centered on well-established facets of experienced EWB—such as mood induction or affective image viewing—adopting standardized measures and validated tasks, could guide the field towards a more unified understanding of the construct and its neural underpinnings.

Alternatively, research into evaluative aspects of EWB may benefit from careful theory-driven task design (e.g., those aimed at understanding the dynamic processes involved in cognitive appraisal of experienced EWB) given the current lack of findings in these areas. Brain imaging measures that allow us to capture these EWB domains dynamically, accounting for their relationship with the environment, could provide crucial insights into the construct and its neural and environmental correlates. Moreover, imaging modalities that allow real-world assessments (i.e., assessments in naturalistic environments) and that are used along with ecological momentary assessments (EMA; Shiffman et al., 2008) would provide the basis for further advancement in the field.

A promising avenue for future research is the use of functional near-infrared spectroscopy (fNIRS). In contrast to the fMRI and EEG modalities used in the included studies, fNIRS offers distinct advantages. Specifically, it demonstrates greater tolerance to bodily movements and boasts heightened portability, compared to MRI, making it well-suited to studies in naturalistic settings (e.g., Hu et al., 2019; Pinti et al., 2020). Researchers can further enhance ecological validity by adopting naturalistic paradigms, such as contrasting neural responses during the viewing of neutral versus emotional films.

Finally, future studies can focus on investigating structural connections in addition to functional connections and interventions to promote EWB or to induce neuroplasticity at grey matter and white matter levels. Innovative approaches that can be incorporated in future studies include the use of multimodal imaging techniques such as fMRI and DTI, or a combination of EEG, fMRI, and DTI. Finally designs that allow investigation of neuromodulation such as TMS-fMRI studies and focused ultrasound (Thaler et al., 2023) are promising.

### 4.3 Brain regions investigated

Although the goal of this review was not to establish neural correlates of EWB, and the search strategy provided a large range of methodologies that make comparison difficult, there were some broad trends in the findings. In general, frontal brain regions are more commonly implicated by studies of positive affect (e.g., van Reekum et al., 2007; Vanderhasselt et al., 2013; Kyeong et al., 2020), while subcortical regions appear more often in studies looking at structural neural correlates of EWB (e.g., Habel et al., 2004; Cunningham and Kirkland, 2014; Lichev et al., 2015; Li et al., 2016). It is difficult to know whether these reflect consistent findings or researchers with different expertise and interest. Similarly, in the temporal domain EEG studies of positive affect mostly targeted alpha frequency but findings related to beta and theta were also reported. Future studies guided by this review to systematically evaluate the neural correlates of EWB should aim to use consistent and well-described neuroimaging methods (Carp, 2012) and share the results (Poldrack et al., 2017) from both whole-brain and ROI analyzes to allow for much needed meta-analyzes (Müller et al., 2018).

## 5 Limitations

### 5.1 Limitations of the studies included in the review

#### 5.1.1 Small sample size

This review identifies several noteworthy limitations within the current body of EWB neuroimaging research. Notably, our analysis revealed that the majority of the studies included relatively small sample sizes. Only 11 of the 95 examined studies included a sample size of 100 or more (e.g., Kujawa et al., 2015; Kong et al., 2015a; Kawamichi et al., 2016). While smaller sample sizes may not compromise interpretation when the observed effect sizes are large, they invariably constrain the power to discern small to medium effects. It is imperative for researchers to judiciously weigh the feasibility and benefits of expanding sample sizes against anticipated effect sizes. It’s also important to acknowledge that advocating for larger sample sizes might serve to exclude researchers from less affluent laboratories globally, potentially perpetuating Western-centric biases in this domain. Furthermore, given the documented volatility of effect sizes within smaller samples (Schönbrodt and Perugini, 2013) and the small to medium effects characteristic of neuroimaging-behavior correlations (Marek et al., 2022; but see Makowski et al., 2023), results from such studies warrant cautious interpretation. Future research would benefit from larger-scale studies and meta-analyzes to bolster confidence in the reported findings.

#### 5.1.2 Lack of studies in diverse samples

As mentioned in previous sections, more than a third of the included studies were conducted in a university setting within healthy individuals. This suggests a probable homogeneity in participants, primarily comprising college students with similar educational backgrounds. While 15.9% of studies delved into EWB within clinical cohorts (e.g., individuals with schizophrenia, those experiencing chronic non-neuropathic back pain, and others), only one study (Martin-Soelch et al., 2020) specifically targeted university students who were kin to a clinical population. Notably absent were studies exploring university students diagnosed with disorders or illnesses; if such participants were included in the studies retained for review, they were not explicitly referenced.

Moreover, our review reveals a conspicuous gap in the literature pertaining to EWB research in children, adolescents, and older adults. This underscores the paucity of dedicated research in these demographics and points to a probable deficiency in the development of validated methodologies and tools tailored for these groups. It is imperative that future endeavors in the field prioritize the selection or development of validated instruments to measure aspects of EWB within these populations. Future studies that consider the impact of life-transition events on emotional well-being are also needed (e.g., middle-aged women hormonal transition and EWB).

Remarkably, none of the studies included in our review presented data on participants’ multifaceted social identities that may bear significance to EWB, such as gender identity, sexual identity, and ability, as highlighted by Villanueva-Moya and Expósito (2022). An intersectional approach to the collection, analysis and reporting of these data would contribute to the collective understand of the diverse elements of identity that contribute to the lived experience of EWB.

#### 5.1.3 Lack of information on race and ethnicity

Another significant limitation observed within the current body of EWB neuroimaging research is the inadequate documentation of participants’ racial and ethnic backgrounds. A mere 9.5% of the articles under review provided details regarding the racial-ethnic demographics of their participants, and this information was exclusively reported by studies based in the United States or Australia.^1^ This underscores a broader oversight in the literature regarding the potential role of racial and ethnic factors on the lived experience of EWB, even in light of considerable research into race-associated stress (refer to Paradies et al. (2015) for an encompassing meta-analysis). We advocate for EWB researchers to exercise heightened diligence in gathering and disclosing race/ethnicity data, while also considering the role these factors play in shaping EWB outcomes. Future research should be committed to inclusivity, ensuring the representation and equitable examination of diverse populations (see National Academies of Sciences, Engineering, and Medicine, 2022 for guidelines). To streamline and elevate global research efforts, there is a need for a consensus on the standardization of racial and ethnic data reporting, recognizing that reporting norms may vary considerably across nations.

#### 5.1.4 Lack of socioeconomic status (SES) information

In addition to the notable absence of racial and ethnic data in the literature, we observed that SES is another demographic characteristic frequently either omitted or incompletely documented. Of the 95 studies included in this review, fewer than 20% provided detailed information on SES (*n* = 17 studies), and among those that did, reporting was typically scant: 16 detailed education, whilst two reported participants’ income. Though several studies implicitly document education level and occupation (e.g., those focusing solely on university student participants), other essential SES metrics, such as family income, remained unreported. The relationship between EWB and SES has been robustly established in prior research (refer to Tan et al. (2020) for a comprehensive meta-analysis). Thus, the importance of SES in the context of EWB studies cannot be overstated, and researchers should ensure it is consistently considered and documented within the context of neural correlates of EWB research.

#### 5.1.5 Lack of causal inference regarding the neural basis of EWB

A significant limitation of the current literature is a paucity of studies designed to elucidate neural basis of EWB. The majority of studies included in this review used a single neuroimaging modality in a cross-sectional design, making causal inferences difficult if not impossible. Many of the longitudinal/intervention studies did not examine links between EWB and neuroimaging measures over time, a missed opportunity to gain improved causal insight into these relationships. Recently, there has been an increased focus on the lack of causal insights in neuroimaging research in general (Siddiqi et al., 2022). We believe that the EWB neuroimaging literature would benefit from Siddiqi and coauthors’ (*Ibid*) recommendations to address this limitation, particularly with respect to the increased use of brain imaging studies that experimentally manipulate the brain (e.g., targeted lesions and stimulation, neurofeedback) and the investigation of temporality (i.e., do brain changes precede changes in EWB). Finally, the literature would benefit from multi-modal studies that can help establish convergence across methodologies to increase confidence of the causal role of certain brain regions in specific aspects of EWB.

### 5.2 Limitations of the present review and future directions

The current findings should be interpreted in light of some limitations specific to this review. First, while we included some studies conducted in Asian countries (e.g., Japan, China), we only included articles that were published in English and mostly in English-speaking countries. As conceptions of EWB differ across cultures (which is highlighted by the working definition used in this study), a more diverse and global approach to this topic is warranted. The growing influence of cultural neuroscience will help to rectify this deficiency in the current literature.

Second, although the range of the included EWB measures provides a wealth of valuable data, the use of self-report scales comes with significant methodological limitations, such as the tendency for self-deception, inability to internalize the relevant features of EWB, and also the subsequent inaccuracy when reporting (e.g., Paulhus and Vazire, 2007; Chan, 2010). Third, we reviewed used studies that included EWB self-report measures that were previously selected by the Koslouski et al. (2022) scoping review of reviews.

Finally, the cultural understanding and interpretation of EWB and its relevant features may differ across cultures and languages. Our inclusion of studies published in English is therefore a limitation. For example, questionnaires in languages other than English might contain content that is difficult to translate into English with fidelity. Moreover, researchers and participants across the globe may have culturally-specific understandings of the concept and essence of EWB [see for example Lomas et al. (2022) and Ruggeri et al., 2020 for global initiatives aiming to understand EWB]. Despite its acknowledged limitations, this scoping review represents an important step towards a better understanding of the neural correlates of EWB, providing an important compilation of the previous studies in this area into one accessible and usable document. The growing influence of cultural neuroscience will be important to address the cultural aspects related to EWB [see Kim and Sasaki (2014) for a review].

Future studies should take a longitudinal approach and consider the complexity of EWB and its relation to a variety of contextual factors (e.g., social, environmental) that could impact an individual’s EWB and their response to interventions. Given its complexity, a team science approach (Hall et al., 2018) is required to allow advancement in the field. The use of multi-level approaches that incorporate multiple imaging modalities in the same study along with multiple behavioral, physiological, and environmental measures would allow us a whole-person integrative approach to investigating EW.B. Finally, a whole person approach (Thomas et al., 2018; Langevin, 2021; Pitcher et al., 2023) is needed to advance the field of EWB.

## Data availability statement

The original contributions presented in the study are included in the article/Supplementary material, further inquiries can be directed to the corresponding author.

## Author contributions

CR: Conceptualization, Data curation, Formal analysis, Investigation, Methodology, Project administration, Supervision, Visualization, Writing – original draft, Writing – review & editing. CL: Data curation, Formal analysis, Investigation, Visualization, Writing – original draft, Writing – review & editing. AT: Conceptualization, Formal analysis, Investigation, Methodology, Writing – original draft, Writing – review & editing. SH: Formal analysis, Investigation, Writing – review & editing. DS: Data curation, Formal analysis, Validation, Writing – review & editing. JL: Formal analysis, Writing – review & editing. DL: Formal analysis, Writing – review & editing. FL: Conceptualization, Funding acquisition, Writing – review & editing. RD: Conceptualization, Funding acquisition, Writing – review & editing. FH: Conceptualization, Funding acquisition, Project administration, Supervision, Writing – review & editing.

## Footnotes

^1^It is plausible that research conducted in nations with more racially homogeneous populations, such as Japan and China, may not prioritize or even collect demographic information, perhaps perceiving it as redundant.
