# Supplementary material for: Brain imaging studies of emotional well-being: a scoping review
Source: Front Psychol. 2024 Jan 5;14:1328523. doi: 10.3389/fpsyg.2023.1328523 (PMC10799564; doi:10.3389/fpsyg.2023.1328523)
Supplement: Supplementary file 1 [file Table_1.pdf]

**Brain Imaging Studies of Emotional Well-Being: A Scoping Review**

**Supplemental Materials**

**Table S1**

*Search Terms Utilized*

| | |
|---|---|
| Group A | emotional well-being OR emotional wellbeing OR psychological well-being OR psychological wellbeing OR subjective well-being OR subjective wellbeing OR life satisfaction OR happiness OR happy OR positive emotion* OR flourish* OR Eudaimoni* OR evaluative well-being OR evaluative wellbeing OR hedonic well-being OR hedonic wellbeing OR experiential well-being OR experiential wellbeing OR spiritual well-being OR spiritual wellbeing OR positive affect OR meaning in life |
| Group B | "magnetic resonance imag*" OR "functional MRI" OR electroencephalogra* OR “event related*” OR event-related* OR "magnetic resonance spectroscop*" OR "positron emission" OR "single-photon emission" OR magnetoencephalogra* OR "Transcranial magnetic stimulation" OR "Transcranial direct current stimulation" OR "diffusion weighted” OR “diffusion-weighted” OR "diffusion tensor" OR “diffusion-tensor” OR “diffusion MRI” OR “diffusion imaging” OR MRI OR fMRI OR EEG OR ERP OR MRS OR PET OR SPECT OR MEG OR TMS OR tDCS OR DWI OR DTI |
| Group C | brain* OR neur* |

*Note.* Group A, B, and C were combined with AND.

**Table S2:** *Full List of Measures Extracted from Articles*

| Measure (Acronym) | Citation |
|---|---|
| Adolescent Quality of Life Instrument (AQoL) | Ward-Smith P, Hamlin J, Bartholomew J, et al. Quality of life among adolescents with cancer. *J Pediatr Oncol Nurs* 2007;24(3):166-71. |
| Affect and Arousal Scale (AFARS) | Chorpita B F, Daleiden E L, Moffitt C, et al. Assessment of tripartite factors of emotion in children and adolescents I. structure validity and normative data of an affect and arousal Scale. *J Psychopathol Behav Assess* 2000; 22(2), 141–160. |
| Affect Balance Scale (ABS) | Bradburn NM, Noll CE. The Structure of Psychological Well-Being. Chicago, IL: Aldine Publishing Company 1969. |
| Affectometer 2 | Kammann R, Flett R. Affectometer 2: a scale to measure current level of general happiness. *Aust J Psychol* 1983;35:259–65. |
| Anamnestic Comparative Self-Assessment Scale (ASCA) | Verhofstadt E, Bleys B, Van Ootegem L. Comparing the anamnestic comparative self-assessment (ACSA) to a conventional happiness question without anchoring. *Appl Res Qual Life* 2019;14: 237–51. |
| Assessment of Quality-of-Life 6D for adolescents (AQOL 6D) | Moodie M, Richardson J, Rankin B, et al. Predicting time trade-off health state valuations of adolescents in four Pacific countries using the Assessment of Quality-of-Life (AQoL-6D) instrument. *Value Health*. 2010;13(8):1014-27. doi: 10.1111/j.1524-4733.2010.00780.x. Epub 2010 Sep 3. PMID: 20825621. |
| Assessment of Quality of Life in Pediatrics (AUQUEI) | Manificat S, Dazord A, Cochat P, et al. Evaluation de la qualité de vie en pédiatric: comment recueillir le point de vue de l'enfant [Evaluation of the quality of life in pediatrics: how to collect the point of view of children]. *Arch Pediatr.* 1997;4(12):1238-46. French. doi: 10.1016/s0929-693x(97)82616-4. PMID: 9538430. |
| Atkinson Life Happiness Rating | Atkinson T. The stability and validity of quality of life measures. *Soc Indic Res* 1982: 10(2), 113-132. |
| Australian Unity Wellbeing Index: Personal Wellbeing Index | Cummins R, Eckersley R, Pallant J, et al. Developing a national index of subjective wellbeing: The Australian Unity Wellbeing Index. *Soc Indic Res* 2003;64(2):159-190. https://doi.org/10.1023/A:1024704320683 |
| BBC Well-being Scale (BSC) | Kinderman P, Schwannauer M, Pontin E, Tai S. The development and validation of a general measure of well-being: the BBC well-being scale. *Qual Life Res* 2011;20(7):1035-42. doi: 10.1007/s11136-010-9841-z. PMID: 21243528. |
| Belgian Subjective Well-Being Scale | Marcoen A, Van Cotthem K, Billiet K, et al. Dimensies van subjectief welbevinden bij ouderen [Dimensions of subjective well-being in elderly persons]. *Tijdschr Gerontol Geriatr* 2002;33(4):156–165. |
| Body-Mind-Spirit Wellness Behavior and Characteristic Inventory (BMS-WBCI) | Hey W, Calderon K, Carroll H. Use of body-mind-spirit dimensions for the development of a wellness behavior and characteristic inventory for college students. *Health Promot Pract* 2006;7(1):125-133. |
| Brief Multi-Dimensional Student Life Satisfaction Scale (BMSLSS) | Seligson J, Huebner E, Valois R. Preliminary validation of the brief multidimensional students' life satisfaction scale (BMSLSS). *Soc Indic Res* 2003;61(2):121-145. |
| Cantril Self-Anchoring Striving Scale (CL) | Cantril H. The Pattern of Human Concerns. New Brunswick, NJ: Rutgers University Press; 1966. |

| | |
|---|---|
| Caregiver Quality of Life Index (CQLI) | McMillan SC, Mahon M. The impact of hospice services on the quality of life of primary caregivers. *Oncol Nurs Forum*. 1994;21:1189-1195. |
| Caregiver Well-being Scale (2nd Version) | Tebb S. An aid to empowerment: a caregiver well-being scale. *Health Soc Work* 1995;20(2):87–92. |
| Caregiver Well-Being Scale: Short Form Rapid Assessment | Tebb SS, Berg-Weger M, Rubio DM. The caregiver well-being scale: developing a short-form rapid assessment instrument. *Health Soc Work* 2013;38(4):222-230. |
| Caregiver Well-Being Scale | Tebb S. An aid to empowerment: A caregiver well-being scale. *Health Soc Work* 1995;20(2):87-92. |
| Caregiver-targeted Quality-of-life measure (CGQOL) | Vickrey B, Hays R, Maines M, et al. Development and preliminary evaluation of a quality of life measure targeted at dementia caregivers. *Health Qual Life Outcomes* 2009;7(56). doi:10.1186/1477-7525-7-56 |
| Carer Well-Being and Support questionnaire (CWS) | Quirk A, Smith SC, Hamilton S, et al. Development of the carer well-being and support (CWS) questionnaire. *Ment Health Rev (Brighton)* 2012;17:128-138. |
| Child and adolescent wellness scale (CAWS) | Copeland EP, Nelson RB, Traughber MC. Wellness dimensions relate to happiness in children and adolescents. *Advances in School Mental Health Promotion*. 2010;3(4):25-37. |
| Chinese Aging Well Profile (CAWP) | Ku PW, Fox KR, McKenna J. Assessing subjective well-being in Chinese older adults: the Chinese aging well profile. *Soc Indic Res*. 2008;87:445-460. |
| Comprehensive Quality of Life Scale-Intellectual Disability (ComQol-ID) | Cummins R. The comprehensive quality of life scale - intellectual disability: an instrument under development. *J Intellect Dev Disabil* 1991;17:2:259-264. |
| Comprehensive Quality of Life Scale | Cummins, R.A. The comprehensive quality of life scale – adult (5th ed.). Melbourne: Deakin University 1997. |
| Con-Dis device for measuring perceived well-being | Reijula J, Rosendahl T, Reijula K, et al. A simple and countable method for the assessment of perceived well-being among elderly people. *International Journal on Smart Sensing and Intelligent Systems* 2017;2(2). |
| Congruity Life Satisfaction Scale | Meadow HL, Mentzer JT, Rahtz DR, et al. A life satisfaction measure based on judgment theory. *Soc Indic Res* 1992;26(1):23-59. |
| Control, Autonomy, Self-realization, Pleasure (CASP-19) | Hyde M, Wiggins RD, Higgs P, et al. A measure of quality of life in early old age: the theory, development and properties of a needs satisfaction model (CASP-19). *Aging Ment Health* 2003;7(3):186-94. |
| Cuestionario de Evaluacion de Calidad de Vida en Contexto Residencial (CECAVIR) | Molero MD, Pérez-Fuentes MD, Gázquez JJ, et al. Construcción y validación inicial de un cuestionario para evaluar la Calidad de Vida en mayores institucionalizados. *Eur J Investig Health Psychol Educ* 2015;2(2):53-65. |
| EPOCH Measure of Adolescent Well-Being (EPOCH) | Kern ML, Benson L, Steinberg EA, et al. The EPOCH measure of adolescent well-being. *Psychol Assess* 2016;28(5):586-97. |
| Escala Bienestar Personal (EBP) | Fierro A, Rando B. Escala eudemon de bienestar personal: características psicométricas [eudemon scale of personal well-being: psychometric characteristics]. *Anuario de Psicología* 2007;38(3): 401–412. |

| | |
|---|---|
| Evaluation of Quality of Life Instrument (EQLI) | Nota L, Soresi S, Perry J. Quality of life in adults with an intellectual disability: the evaluation of quality of life instrument. *J Intellect Disabil Res* 2006;50(5):371-85. |
| Extended Satisfaction With Life Scale (ESWLS) | Alfonso VC, Allison DB, Rader DE, et al. The extended satisfaction with life scale: development and psychometric properties. *Soc Indic Res* 1996;38(3):275-301. |
| Family Caregiver of Life Scale (FAMQOL) | Nauser JA, Bakas T, Welch JL. A new instrument to measure quality of life of heart failure family caregivers. *J Cardiovasc Nurs* 2011;26(1):53-64. |
| Ferrans and Powers Quality of Life Index (Ferrans and Powers QLI) | Ferrans CE, Powers MJ. Quality of life index: development and psychometric properties. *ANS Adv Nurs Sci* 1985;8:15-24. |
| Five-Factor WEL (5F-Wel) | Hattie JA, Myers JE, Sweeney TJ. A factor structure of wellness: Theory, assessment, analysis, and practice. *J Couns Dev* 2004;82(3):354-64. |
| Four-Factor WEL (4F-WEL) | Myers JE, Luecht RM, Sweeney TJ. The factor structure of wellness: reexamining theoretical and empirical models underlying the wellness evaluation of lifestyle (WEL) and the five-factor Wei. *Meas Eval Couns Dev* 2004;36(4):194-208. |
| Friedman Well-Being Scale | Friedman PH. Friedman well-being scale and professional manual, foundation for well–being. *Eur Rev Soc Psychol* 1992;4:1-26. |
| Gallup Healthways Well-Being Index (WBI) | Harter JK, Gurley VF. Measuring well-being in the United States. *APS Observer* 2008;21(8). |
| General Well-being Schedule (GWB) | Dupuy HJ. The General Well-Being Schedule. In McDowell I, Newell C, eds. Measuring Health: A Guide to Rating Scales and Questionnaire (2nd ed). New York City, NY: Oxford University Press 1977;206-13. |
| Generic Children's Quality of Life Measure (GCQ) | Collier J, MacKinlay D, Phillips D. Norm values for the generic children's quality of life measure (GCQ) from a large school-based sample. *Qual Life Res* 2000;9(6):617-23. |
| Gothenburg Quality of Life Measurement Scale (GQL) | Tibblin G, Tibblin B, Peciva S, et al. " The göteborg quality of life instrument"--an assessment of well-being and symptoms among men born 1913 and 1923. methods and validity. *Scand J Prim Health Care Suppl* 1990;1:33-8. |
| How Are You? (HAY) | Maes S, Bruil J. Assessing the quality of life in children with a chronic illness. In: Rodriguez-Marin J, ed. Health Psychology and Quality of Life Research. Alicante, Spain: Health Psychology Department, University of Alicante; 1995;637–52. |
| ICEpop CAPability measure for Adults (ICECAP-A) | Al-Janabi H, Flynn TN, Coast J. Development of a self-report measure of capability wellbeing for adults: the ICECAP-A. *Qual Life Res* 2012;21(1):167-76. |
| ICEpop CAPability measure for older people (ICECAP-O) | Coast J, Peters TJ, Natarajan L, et al. An assessment of the construct validity of the descriptive system for the ICECAP capability measure for older people. *Qual Life Res* 2008;17(7):967-76. |
| Index of General Affect | Campbell A. Subjective measures of well-being. *American Psychologist.* 1976;31(2):117-24. |

| | |
|---|---|
| Infant and Toddler Quality of Life Questionnaire (ITQOL) | Landgraf JM, Abetz L. The infant/toddler quality of life questionnaire: conceptual framework, logic, content, and preliminary psychometric results. final report to Schering-Plough Laboratories and Health Technology Associates. Boston: New England Medical Center. 1994. |
| Integration Inventory (II) | Ruffing-Rahal MA. Initial psychometric evaluation of a qualitative well-being measure: the integration inventory. *Health Values* 1991;15(2):10-20. |
| Interactive Computerized Quality of life Scale-SF (ICQOL-SF) | Jamison RN, Fanciullo GJ, McHugo GJ, et al. Validation of the short-form interactive computerized quality of life scale (ICQOL-SF). *Pain Med* 2007;8(3):243-50. |
| Koskenvuo Quality of Life Scale | Kaprio J, Koskenvuo M, Artimo M, et al. The Finnish Twin Registry: Baseline Characteristics. Section I. Materials, Methods, Representativeness and Results for Variables Special to Twin Studies. Helsinki: Helsingin yliopiston monistuspalvelu, 1979. |
| Kuwaiti Raha Scale (KRS) | Ridha H, Al Naser F, Figley CR. Developing a measure of contentment in an arab muslim country: implications for cross-cultural research in social work. *European Journal of Social Work* 2008;11(4):459-67. |
| Lancashire Quality of Life Profile-modified (LQoLP-modified) | Van Nieuwenhuizen C, Schene AH, Koeter MW, et al. The lancashire quality of life profile: modification and psychometric evaluation. *Soc Psychiatry Psychiatr Epidemiol* 2001;36(1):36-44. |
| Life Dimensions Questionnaire (LDQ-30) | Roberts RE, Pascoe GC, Attkisson CC. Relationship of service satisfaction to life satisfaction and perceived well-being. *Eval Program Plann* 1983;6(3-4):373-83. |
| Life Orientation Test-Revised (LOT-R) | Scheier MF, Carver CS, Bridges MW. Distinguishing optimism from neuroticism (and trait anxiety, self-mastery, and self-esteem): a reevaluation of the life orientation test. *J Pers Soc Psychol* 1994;67(6):1063-78. |
| Life Orientation Test (LOT) | Scheier MF, Carver CS. Optimism, coping, and health: assessment and implications of generalized outcome expectancies. *Health Psychology* 1985;4(3):219-47. |
| Life Orientation Test of Optimism and Pessimism - Revised (LOT-R) | Scheier MF, Carver CS, Bridges MW. Distinguishing optimism from neuroticism (and trait anxiety, self-mastery, and self-esteem): a reevaluation of the Life Orientation Test. *J Pers Soc Psychol* 1994;67:1063–78. |
| Life Satisfaction Index | Neugarten BL, Havighurst RJ, Tobin SS. The measurement of life satisfaction. *J Gerontol* 1961. |
| Life Satisfaction Index Third Age (LSITA) | Barrett AJ, Murk PJ. Life Satisfaction Index for the Third Age (LSITA): A Measurement of Successful Aging. In Isaac EP, ed. Proceedings of the 2006 Midwest Research-to-Practice Conference in Adult, Continuing, and Community Education. St. Louis, MO: University of Missouri 2006:7-12. |
| Life Satisfaction Matrix | Lyons G. The life satisfaction matrix: an instrument and procedure for assessing the subjective quality of life of individuals with profound multiple disabilities. *J Intellect Disabil Res* 2005;49(10):766-9. |
| Life Satisfaction Scale (LSS) | Heal LW, Chadsey-Rusch J. The lifestyle satisfaction scale (LSS): assessing individuals' satisfaction with residence, community |

| | |
|---|---|
| | setting, and associated services. *Appl Res Ment Retard* 1985;6(4):475-90. |
| Lifestyle Assessment Questionnaire (LAQ) | Mackie PC, Jessen EC, Jarvis SN. The Lifestyle Assessment Questionnaire (LAQ-CP) Manual. Newcastle upon Tyne: North of England Collaborative Cerebral Palsy Survey 1998:2-5. |
| Life Satisfaction Index (LSI) | Neugarten BL, Havighurst RJ, Tobin SS. The measurement of life satisfaction. *J Gerontol* 1961;16:134-43. |
| Linear Analog Self-Assessment | Locke DE, Decker PA, Sloan JA, et al. Validation of single-item linear analog scale assessment of quality of life in neuro-oncology patients. *J Pain Symptom Manage* 2007;34(6):628-638. doi:10.1016/j.jpainsymman.2007.01.016 |
| Manchester Short Assessment of Quality of Life (MANSA) | Priebe S, Huxley P, Knight S, et al. Application and results of the manchester short assessment of quality of life (MANSA). *Int J Soc Psychiatry* 1999;45(1):7-12. |
| Maryland Ask Me! Project (MAMP) | Bonham GS, Basehart S, Schalock RL, et al. Consumer-based quality of life assessment: the Maryland ask me! project. *Ment Retard* 2004;42(5):338-55. |
| McGill Quality of Life Questionnaire (MQOL) | Cohen SR, Mount BM, Strobel MG, et al. The mcgill quality of life questionnaire: a measure of quality of life appropriate for people with advanced disease. a preliminary study of validity and acceptability. *Palliat Med* 1995;9(3):207-19. |
| Meaning in Life Questionnaire (MLQ) | Steger MF, Frazier P, Oishi S, et al. The meaning in life questionnaire: assessing the presence of and search for meaning in life. *J Couns Psychol* 2006;53(1):80-93. |
| Meaning in Life Scale (MLS) | Lee SE, Hong, G. Development of the meaning in life scale for older adults. *J Korean Acad Nurs* 2017;47(1):86–97. |
| Mental Health Continuum-Short Form (MHC-SF) | Lamers SMA, Westerhof GJ, Bohlmeijer ET, et al. Evaluating the psychometric properties of the mental health continuum-short form (MHC-SF). *J Clin Psychol*. 2011;67(1):99–110. |
| Mental Health Inventory (MHI) | Veit CT, Ware JE. The structure of psychological distress and well-being in general populations. *J Consult Clin Psychol* 1983;51(5):730-42. |
| Modular System for Quality of Life (MSQOL) | Pukrop R, Möller HJ, Steinmeyer EM. Quality of life in psychiatry: a systematic contribution to construct validation and the development of the integrative assessment tool "modular system for quality of life". *Eur Arch Psychiatry Clin Neurosci* 2000;250(3):120-32. |
| Multicultural Quality of Life Index (MQLI) | Mezzich JE, Cohen NL, Ruiperez MA, et al. The multicultural quality of life index: presentation and validation. *J Eval Clin Pract* 2011;17(2):357-64. |
| Multidimensional Personality Questionnaire Well-being Scale (MPQ-WB) | Sellbom M, Ben-Porath, YS. Mapping the MMPI-2 restructured clinical scales onto normal personality traits: evidence of construct validity. *J Pers Assess* 2005;85(2):179–87. |
| Multi-dimensional Student Life Satisfaction Scale (MSLSS) | Huebner ES. Preliminary development and validation of a multidimensional life satisfaction scale for children. *Psychol Assess* 1994;6(2):149-58. |
| Multifaceted Life Satisfaction Scale (MLSS) | Harner CJ, Heal LW. The multifaceted lifestyle satisfaction scale (MLSS): Psychometric properties of an interview schedule for assessing personal satisfaction of adults with limited intelligence. *Res Dev Disabil* 1993;14(3):221-36. |

| | |
|---|---|
| Nordic Quality of Life Questionnaire for Children | Lindström B, Eriksson B. Quality of life among children in the Nordic countries. *Qual Life Res* 1993;2(1):23-32. |
| Older People's Quality of Life questionnaire (OPQOL) | Bowling A. The psychometric properties of the older people's quality of life questionnaire, compared with the CASP-19 and the WHOQOL-OLD. *Curr Gerontol Geriatr Res* 2009; 298950. |
| Orientation to Happiness Scale (OTH) | Peterson C, Park N, Seligman ME. Orientations to happiness and life satisfaction: the full life versus the empty life. *J Happiness Stud* 2005;6(1):25-41. |
| Positive and Negative Affect Scale - child version (PANAS-C) | Laurent J, Catanzaro SJ, Joiner Jr TE, et al. A measure of positive and negative affect for children: scale development and preliminary validation. *Psychol Assess* 1999;11(3):326-338. |
| Pediatric Quality of Life Enjoyment and Satisfaction Questionnaire (PQ-LES-Q) | Endicott J, Nee J, Yang R, et al. Pediatric quality of life enjoyment and satisfaction questionnaire (PQ-LES-Q): reliability and validity. *J Am Acad Child Adolesc Psychiatry* 2006;45(4):401-7. |
| Perceived Life Satisfaction Scale (PLSS) | Adelman HS, Taylor L, Nelson P. Minors' dissatisfaction with their life circumstances. *Child Psychiatry Hum Dev* 1989;20(2):135-47. |
| Perceived Well-Being Scale (PWB) | Reker GT, Wong PT. Psychological and physical well-being in the elderly: the perceived well-being scale (PWB). *Can J Aging* 1984;3(1):23-32. |
| Perceived Wellness Survey (PWS) | Adams T, Bezner J, Steinhardt M. The conceptualization and measurement of perceived wellness: integrating balance across and within dimensions. *Am J Health Promot* 1997;11(3):208-18. |
| Personal Outcome Scale (POS) | Van Loon J, Van Hove G, Schalock R, et al. Personal Outcomes Scale: Administration and Standardization Manual. Ghent, Netherlands: Stichting Arduin. 2009. |
| PGI well-being scale (PGI) | Verma SK, Dubey BL, Gupta D. P.G.I. general wellbeing scale. *Indian J Clin Psychol* 1983;10:299–304. |
| Physiological Measure of Subjective Well-Being of Persons with Profound Intellectual or Multiple Disabilities | Vos P, De Cock P, Petry K, et al. Do you know what I feel? A first step towards a physiological measure of the subjective well-being of persons with profound intellectual and multiple disabilities. *J Appl Res Intellect Disabil* 2010;23(4):366-78. |
| Pictures Child's Quality of Life Self Questionnaire (AUQUEI) | Magnificat S, Dazord A. Children's quality of life assessment: preliminary results obtained with the AUQUEI questionnaire. *QOL Newsletter* 1998;15:2–3. |
| Positive and Negative Affect Schedule (PANAS) | Watson D, Clark LA, Tellegen A. Development and validation of brief measures of positive and negative affect: the PANAS scales. *J Pers Soc Psychol* 1988;54(6):1063-1070. |
| Positive Mental Health Measurement Scale (PMH) | Vaingankar JA, Subramaniam M, Chong SA, et al. The positive mental health instrument: development and validation of a culturally relevant scale in a multi-ethnic Asian population. *Health Qual Life Outcomes* 2011;9(1):1-8. |
| Positive Valuation of Life Scale (VoL) | Lawton MP, Moss M, Hoffman C, et al. Valuation of life: a concept and a scale. *J Aging Health* 2001;13(1):3-31. |
| Public Health Surveillance Well-being Scale (PHS-WB) | Bann CM, Kobau R, Lewis MA, et al. Development and psychometric evaluation of the public health surveillance well-being scale. *Qual Life Res* 2012;21(6):1031-43. |

| | |
|---|---|
| QUALIDEM | Ettema TP, Dröes RM, De Lange J, et al. QUALIDEM: development and evaluation of a dementia specific quality of life instrument. scalability, reliability and internal structure. *Int J Geriatr Psychiatry* 2007;22(6):549-56. |
| QUALIN | Manificat S, Dazord A, Langue J, et al. Evaluation de la qualite de vie du nourrisson et du tres jeune enfant: validation d'un questionnaire. Etude multicentrique europeenne (Assessing infant's quality of life: validation of a new questionnaire. A multicentric European study). *Arch Pediatrie* 2000;7:605e14. |
| Quality of Life Assessment Form | McGuire BE, Choon G, Akuffo E. Community living for elderly people with an intellectual disability: a pilot study. *J Intellect Dev Disabil* 1991;17(1):25-33. |
| Quality of Life Assessment Schedule (QOLAS) | Selai CE, Trimble MR, Rossor MN, et al. Assessing quality of life in dementia: preliminary psychometric testing of the quality of life assessment schedule (QOLAS). *Neuropsychol Rehabil* 2001;11(3-4):219-43. |
| Quality of Life Assessment Tool | Johnson R, Cocks H. Quality of Life: An Assessment Strategy. Users Manual. Challenge Foundation, Armidale. 1989. |
| Quality of Life, Enjoyment and Satisfaction Questionnaire (Q-LES-Q) | Endicott J, Nee J, Harrison W, et al. Quality of life enjoyment and satisfaction questionnaire: a new measure. *Psychopharmacol Bull* 1993;29(2):321-6. |
| Quality of Life Index | Padilla GV, Presant C, Grant MM, et al. Quality of life index for patients with cancer. *Res Nurs Health* 1983;6(3):117–26. |
| Quality of Life Instrument | Janssen CG, Vreeke GJ, Resnick S, et al. Quality of life of people with mental retardation–residential versus community living. *The British Journal of Developmental Disabilities* 1999;45(88):3-15. |
| Quality of Life Interview Schedule (QUOLIS) | Ouellette-Kuntz H. A pilot study in the use of the quality of life interview schedule. *Soc Indic Res* 1990;23(3):283-98. |
| Quality of Life Interview-Brief Version (QOLI-BV) | Lehman AF. A quality of life interview for the chronically mentally ill. *Eval Program Plann* 1988;11(1):51-62. |
| Quality of Life Inventory (QOLI) | Frisch MB, Cornell J, Villanueva M, et al. Clinical validation of the quality of life inventory. a measure of life satisfaction for use in treatment planning and outcome assessment. *Psychol Assess* 1992;4(1):92-101. |
| Quality of Life Profile Adolescent Version | Raphael D, Rukholm E, Brown I, et al. The quality of life profile—adolescent version: background, description, and initial validation. *J Adolesc Health* 1996;19(5):366-75. |
| Quality of Life Questionnaire | Brown RI, Bayer MB. Rehabilitation Questionnaire Manual: A Personal Guide to the Individual's Quality of Life: A Review of the Consumer's Perspective. Captus Press; 1992. |
| Quality of My Life questionnaire (QoML) | Feldman BM, Grundland B, McCullough L, et al. Distinction of quality of life, health related quality of life, and health status in children referred for rheumatologic care. *J Rheumatol* 2000; 27: 226–33. |
| Ryffs Scales of Psychological Well-Being-18 (RPWB) | Ryff CD, Keyes CL. The structure of psychological well-being revisited. *J Pers Soc Psychol* 1995;69(4):719-27. |

| | |
|---|---|
| Ryffs Scales of Psychological Well-Being-54 (RPWB) | Ryff CD, Keyes CL. The structure of psychological well-being revisited. *J Pers Soc Psychol* 1995;69(4):719-27. |
| Ryffs Scales of Psychological Well-Being-84 (RPWB) | Ryff CD, Keyes CL. The structure of psychological well-being revisited. *J Pers Soc Psychol* 1995;69(4):719-27. |
| Salamon-Conte Life Satisfaction in the Elderly Scale (SCLSES) | Conte VA, Salamon MJ. An objective approach to the measurement and use of life satisfaction with older persons. *Measurement and Evaluation in Guidance* 1982;15(3):194-200. |
| Satisfaction With Life Scale (SWLS) | Pavot W, Diener E. Review of the satisfaction with life scale. *Psychol Assess* 1993;5(2):164–72. |
| Scale of Happiness of the Memorial University of Newfoundland (MUNSH) | Kozma A, Stones MJ. The measurement of happiness: Development of the Memorial University of Newfoundland Scale of Happiness (MUNSH). *J Gerontol* 1980;35(6):906-12. |
| Schedule for Evaluation of Individual QOL-Direct Weight (SEIQOL-DW) | O'Boyle CA. The schedule for the evaluation of individual quality of life (SEIQoL): the concept of quality of life in clinical research. *Int. J. Ment. Health* 1994;23(3):3-23. |
| Self-evaluation of Quality of Life (SEQOL) | Ventegodt S, Merrick J, Andersen NJ. Measurement of quality of life III. From the IQOL theory to the global, generic SEQOL questionnaire. *ScientificWorldJournal* 2003;3:972-91. |
| Sense of Coherence Scale (SOC) | Antonovsky A. The structure and properties of the sense of coherence scale. *Soc Sci Med* 1993;36(6):725-33. |
| Sense of Well-Being Inventory (SWBI) | Rubin SE, Chan F, Bishop M, et al. Psychometric validation of the sense of well-being inventory for programme evaluation in rehabilitation. *Professional Rehabilitation* 2003;11:54– 59. |
| Social and Emotional Well-Being Module | Australian Institute of Health Welfare. Measuring the social and emotional wellbeing of Aboriginal and Torres Strait Islander peoples. Canberra: Australian Institute of Health and Welfare. 2009. http://www.aihw.gov.au/publication-detail/?id=6442468208 (accessed 7 Feb 2022). |
| Social and Emotional Health Survey (SEHS) | Furlong MJ, You S, Renshaw TL, et al. Preliminary development and validation of the social and emotional health survey for secondary school students. *Soc Indic Res* 2014;117(3):1011-32. |
| SPF-IL | Nieboer A, Lindenberg S, Boomsma A, et al. Dimensions of well-being and their measurement: the SPF-IL scale. *Soc Indic Res* 2005;73(3):313-53. |
| Strong Souls | Thomas A, Cairney S, Gunthorpe W, et al. Strong souls: development and validation of a culturally appropriate tool for assessment of social and emotional well-being in Indigenous youth. *Aust N Z J Psychiatry* 2010;44(1):40-8. |
| Student Life Satisfaction Scale (SLSS) | Huebner ES. Initial development of the student's life satisfaction scale. *Sch Psychol Int* 1991;12(3):231-240. |
| Subjective Happiness Scale (SHS) | Lyubomirsky S, Lepper HS. A measure of subjective happiness: preliminary reliability and construct validation. *Soc Indic Res* 1999;46(2):137-55. |
| Subjective Quality of Life Profile (SQLP) | Dazord A, Gerin P, Boissel JP. Subjective quality of life assessment in therapeutic trials: presentation of a new instrument in France (SQLP: subjective quality of life profile) and first results. *Quality of Life Assessment: International Perspectives* 1994;185–95. |

| | |
|---|---|
| Thai Elders Psychological Well-Being measure | Ingersoll-Dayton B, Saengtienchai C, Kespichayawattana J, et al. Measuring psychological well-being: insights from Thai elders. *Gerontologist* 2004;44(5):596-604. |
| Thriving of Older People Assessment Scale (TOPAS) | Bergland Å, Kirkevold M, Sandman PO, et al. The thriving of older people assessment scale: validity and reliability assessments. *J Adv Nurs* 2015;71(4):942-51. |
| Valuation Of Life Scale (VOL) | Lawton MP, Moss M, Hoffman C, et al. Valuation of life: a concept and a scale. *J Aging Health* 2001;13(1):3-31. |
| Visual Analog Scale on Quality of Life (VAS) | Scott J, Huskisson EC. Graphic representation of pain. *Pain* 1976;2(2):175–84. |
| Warwick-Edinburgh Mental Well-Being Scale (WEMWBS) | Tennant R, Hiller L, Fishwick R, et al. The Warwick-Edinburgh mental well-being scale (WEMWBS): development and UK validation. *Health Qual Life Outcomes* 2007;5(63). https://doi.org/10.1186/1477-7525-5-63 |
| Warwick-Edinburgh Mental Well-Being Scale Short Version (SWEMWBS) | Stewart-Brown S, Tennant A, Tennant R, et al. Internal construct validity of the Warwick-Edinburgh mental well-being scale (WEMWBS): A rasch analysis using data from the Scottish health education population survey. *Health Qual Life Outcomes* 2009;7:1–8. |
| Well-Being Questionnaire (W-BQ) | Bradley C. The 12-item well-being questionnaire: origins, current stage of development, and availability. *Diabetes Care* 2000;23:875 |
| Wellness Evaluation of Lifestyle (WEL) | Myers JE, Sweeney TJ, Witmer JM. The wheel of wellness counseling for wellness: a holistic model for treatment planning. *J Couns Dev* 2000;78(3):251-66. |
| Wellness Index | Slivinske LR, Fitch VL, Morawski DP. The wellness index: developing an instrument to assess elders' well-being. *J Gerontol Soc Work* 1996;25(3-4):185-204. |
| WHO Quality of Life Scale - Older population (WHOQOL-OLD) | Power M, Quinn K, Schmidt S. Development of the WHOQOL-old module. *Qual Life Res* 2005;14(10):2197-214. |
| WHO-5 Well-being Index (WHO-5) | Topp CW, Østergaard SD, Søndergaard S, et al. The WHO-5 well-being index: a systematic review of the literature. *Psychother Psychosom* 2015;84(3):167-76. |
| WHOQOL-100 | Power M, Kuyken W, Orley J, et al. The World Health Organization quality of life assessment (WHOQOL): development and general psychometric properties. *Soc Sci Med* 1998;46(12):1569-85. |
| WHOQOL-Bref | The Whoqol Group. The World Health Organization quality of life assessment (WHOQOL): development and general psychometric properties. *Soc Sci Med* 1998;46(12):1569-85. |
| Youth Quality of Life instrument - Research version (YQoL-R) | Patrick DL, Edwards TC, Topolski TD. Adolescent quality of life, part II: initial validation of a new instrument. *J Adolesc* 2002;25(3):287-300. |

*Note*. This table was adapted from Koslouski et al. (2022)

**Table S3**

Aims and Findings of Studies Included for Analysis (N=95)

| First Author (Year) | Aims | Conclusions |
|---|---|---|
| Alessandri (2015) | To investigate cortical structures associated with personality dimension of positivity (POS) by using a sLORETA. | POS is a basic disposition that reflects the concerted activity of brain structures that are essential for integrating self-referential thought and autobiographical memories and for assigning a positive valence to one’s experience and attitude toward the future. |
| Arjmand (2017) | Aims to examine whether: (1) music perceived as, emotionally powerful, and pleasant by listeners also elicited a response in a central marker of emotional response (frontal alpha asymmetry); and (2) peaks in frontal alpha asymmetry were associated with changes in key musical or psychoacoustic events associated with emotion. | Significant effects in frontal asymmetry (FA) were observed in the frontal electrode pair FC3– FC4, and the greatest increase in left bias from baseline was observed in response to pleasurable music. Peak FA responses at this site were also found to co-occur with key musical events relating to change, for instance, the introduction of a new motif, or an instrument change, or a change in low level acoustic factors such as pitch, dynamics or texture. |
| Baeken (2008) | To replicate previous HF-rTMS findings on the left dlPFC in a new (large) group of healthy female subjects with a focus especially on positive mood changes and extend the former research by stimulating the right dlPFC in a different but comparable (large) group of healthy female volunteers with the same HF-rTMS parameters. | Unable to demonstrate immediate or delayed mood changes after one single active HF-rTMS session on the left or right dlPFC. Although authors took into account several methodological problems which might have confounded previous rTMS mood induction studies, the hypothesis that one single session of HF-rTMS on the left or on the right dlPFC can influence mood in healthy female volunteers was not supported. |
| Costa (2019) | To investigate the neural correlates of both hedonic and eudaimonic happiness. | Hedonic and eudaimonic happiness activate similar neural correlates. However, both kinds of happiness are also associated with distinctive brain areas serving distinctive functions. |
| Cunningham (2014) | Investigate the mechanisms of happiness and well-being relative to the neural process involved in depression/anxiety. | Happier participants showed greater amygdala responses to positive stimuli. Moreover, no significant relationships were found between happiness and responses to negative stimuli. In other words, for happy people, a tuning toward positive did not come at the cost of losing sensitivity to negativity. This work suggests that trait happiness is associated with a balanced amygdala response to positivity and negativity. |
| Davidson (2003) | To study the underlying changes in biological processes that are associated with reported changes in mental and physical health in response to meditation. | A short program in mindfulness meditation produces demonstrable effects on brain and immune function. These findings suggest that meditation may change brain and immune function in positive ways and underscore the need for additional research. |
| Day (2019) | To evaluate the behavioral inhibition and activation system (BIS-BAS) model of pain. | This is the first study to test these associations from the perspective of a BIS-BAS model of pain. The |

| | | |
|---|---|---|
| | Frontal alpha asymmetry (FAA) as a possible neurophysiological correlate of the BIS-BAS was also explored, as was the role of personality factors. | findings provide preliminary support for the central tenets of this framework. |
| Dennison (2015) | To prospectively explore the relationship between trait indices of PA and brain development in subcortical reward regions during early to mid-adolescence in a community sample of adolescents. | Early to mid-adolescence is an important period whereby neurodevelopmental processes may underlie key phenotypes conferring both risk and resilience for emotional and behavioral difficulties later in life. |
| Dolcos (2021) | To test a novel cognitive-emotional training intervention targeting the development of emotion regulation skills aimed at increasing resilience against emotional distress. | Resilience and well-being can be learned through emotion regulation training, and that training-related improvements manifested in both behavioral change and neuroplasticity can translate into real-life benefits. |
| Flores (2018) | To investigate whether neural response to social reward (1) is related to the experience of emotional closeness and (2) moderates the association between emotional closeness and PA during and following social interactions. | Demonstrate the importance of neural response to social reward in key social processing regions for everyday experiences of emotional closeness and PA in the context of social interactions. |
| Fonzo (2017) | To assess changes in brain function after prolonged exposure therapy across three emotional reactivity and regulation paradigms. | Changes in frontopolar function during de- liberate regulation of NA is one key mechanism of adaptive psychotherapeutic change in PTSD. Given that frontopolar connectivity with ventromedial regions during emotion regulation is enhanced by psychotherapy and that the frontopolar cortex exerts downstream influence on ventromedial regions in healthy individuals, these findings inform a novel conceptualization of how psychotherapy works, and they identify a promising target for stimulation-based therapeutics |
| Geethanjali (2019) | To investigate FC of emotional responses to Indian Dharmavathi raga music. | Findings indicate that Indian Dharmavathi raga induces a positive emotion. The relative theta band energy at frontal locations was increased during Task (listening to Dharmavathi raga) as compared with Rest (silence) condition. The relative energy of beta band at frontal locations was significantly decreased after listening to raga. The raga played evoked an increased bi-frontal theta response, which indicates that raga is pleasant and induces mood change after listening. |
| George (1995) | To determine if H2150 PET might demonstrate changes in regional cerebral blood flow (rCBF) associated with transient sadness or happiness in healthy adult women. | Transient sadness and happiness in healthy volunteer women are accompanied by significant changes in regional brain activity in the limbic system, as well as other brain regions. Transient sadness and happiness affect different brain regions in divergent directions and are not merely opposite activity in identical brain regions. These findings have implications for understanding the neural substrates of both normal and pathological emotion. |
| Gondoh (2009) | To examine the effects of aerobic exercise training on brain structure and PWB in young adults. | Aerobic exercise training may inhibit GM volume loss in the insula, and that a relationship may exist between preservation of insula GM and improvement of PWB by aerobic exercise training. |
| Habel (2004) | To assess the genetic influence of negative mood experience in patients with schizophrenia. | Mood induction was successful in all groups according to subjective ratings. During sadness induction, the patients and their nonaffected siblings demonstrated less activity in the amygdala compared with the healthy group of nonrelatives. Other regions of interest, such as the left OFC, the left superior temporal cortex, and the left precuneus/posterior cingulate revealed significant group differences only between patients and nonrelated healthy subjects. During positive mood induction, |

| | | |
|---|---|---|
| | | no group differences could be found in the amygdala. |
| Hagemann (1999) | (1) to test the association between anterior asymmetry and dispositional mood as predicted by the model of anterior asymmetry and emotion and (2) to investigate if the model might be extended to extraversion and neuroticism. | Subjects who scored high on NA had greater relative left-sided cortical activation than subjects scoring low on NA which was due to greater absolute activation of the left anterior temporal site in high NA scorers. There were no associations between asymmetry and PA. A functional neuroanatomical model is presented which suggests that greater tonical activation of the left temporal cortex increases the susceptibility to experience negative emotions. |
| Hall (1999) | To examine the relationship between physical activity, frontal asymmetry, and dispositional affect in older adults. | The relationship between frontal brain activity and dispositional affect is influenced by physical activity in older adults. |
| Hasler (2012) | To explore and compare diurnal patterns of PA and the activity of PA regions in morning- and evening-types with insomnia. | Alterations in the diurnal activity of PA-related neural structures may underlie differences in the phase and amplitude of self-reported PA and evening chronotypes, and may constitute one mechanism for increased risk of mood disorders among evening- type insomniacs. |
| He (2019) | To characterize the dysconnectivity pattern within this system and explore the relationships between this kind of dysconnectivity pattern and affective symptoms, which might help us better look into the neuro pathophysiological mechanisms underlying major depressive disorders (MDD). | Demonstrated that MDD showed characteristic pathological alterations of the emotion regulation system. Dysconnectivity within prefrontal–limbic system might be more related to the dysregulation of NA, whereas dysconnectivity within prefrontal–striatum system might influence more on PA processing. The decrease in PA and increase in NA in MDD might have different pathological basis. These results could help better understand the dysconnectivity pattern in the emotion-regulating system underlying depression. |
| Heller (2013a) | To determine whether treatment-induced changes in the ability to sustain nucleus accumbens activity and fronto-striatal connectivity during the regulation of PA are associated with gains in tPA. | Treatment-induced change in the sustained engagement of fronto-striatal circuitry tracks the experience of positive emotion in daily life. Studies examining reduced PA in a variety of psychiatric disorders might benefit from examining the temporal dynamics of brain activity when attempting to understand changes in daily PA. |
| Heller (2013b) | To examine whether individual differences in the sustained engagement of reward circuitry in response to affective stimuli were related to health and well-being. | Individuals with sustained activity in the striatum and dlPFC to positive stimuli over the course of the scan session reported greater well-being and had lower cortisol output. This suggests that sustained engagement of reward circuitry in response to positive events underlies well-being and adaptive regulation of the hypothalamic-pituitary-adrenal axis. |
| Hiyoshi-Taniguchi (2013) | To induce a controlled perturbation in the emotional system of the brain by multimodal stimuli, and to investigate whether such emotional stimuli could induce reproducible and consistent changes in EEG signals. | Demonstrated that EEG could be used as a tool to investigate emotional valence and discriminate various emotions. |
| Hofer (2007) | To investigate gender differences in regional cerebral activity during the perception of positive or negative emotions using event-related fMRI. | Gender-related neural responses to emotional stimuli and could contribute to the understanding of mechanisms underlying the gender disparity of neuropsychiatric diseases such as mood disorders. |
| Isbel (2019) | To investigate the underlying neurobiological changes supporting these outcomes by assessing anterior alpha asymmetry. | Results showed that 8 weeks of mindfulness training is capable of inducing changes in resting anterior alpha asymmetry, but these results are dependent upon the level of proficiency achieved. |

| | | |
|---|---|---|
| Jung (2002) | To determine the relationship between Cho and mood in a cohort of healthy young subjects. | Highlighted the important involvement of choline (Cho) underlying the integration of affective processing within prefrontal circuitry, and may indicate increased myelin turnover in subjects with lower PA. Further efforts will be necessary to determine if high Cho is associated with increased incidence of mood disorders throughout life. |
| Katsumi (2021) | To reveal the neural mechanisms of subjective happiness and trait empathy in a sample of young females with a data-driven, multi-voxel pattern analysis of whole-brain intrinsic FC. | Results identified an important contribution of the macroscale functional organization of the brain to human well-being, by demonstrating that lower levels of personal distress led to higher subjective happiness through variation in intrinsic FC along a neural representation vs. modulation gradient. |
| Kawamichi (2016) | To compare subjective happiness, an indirect measure of the existence of positive experiences caused by being in a romantic relationship, of participants with or without romantic partners. | Being in a romantic relationship enhances perceived subjective happiness via positive experiences. Furthermore, the observed reduction in GM density in the right dorsal striatum may reflect an increase in saliency of social reward within a romantic relationship. Thus, being in a romantic relationship is associated with positive experiences and a reduction of GM density in the right dorsal striatum, representing a modulation of social reward. |
| Killgore (2007) | To examine the relationship between affect ratings on the PANAS and activity within the occipital cortex of 13 normal-weight women while viewing images of high calorie and low calorie foods using fMRI. | Findings suggest a mechanism whereby PA state may affect the early stages of sensory processing, possibly influencing subsequent perceptual experience of a stimulus. |
| Koepp (2009) | To demonstrate, consistent with Panksepp's hypothesis, that experience of positive emotions is associated with decreased amygdala activity modulated through increased opioid-related inhibition. | The finding of reduced [$^{11}$C] diprenorphine binding is consistent with increased release of endogenous opioids, providing direct evidence that localized release of endogenous opioids is involved in the regulation of positive emotion in humans. |
| Kohn (2014) | To investigate the relationship between brain activity and inter-individual variation in subjective affect ratings. | Finding is in line with previous observations of the subgenual cingulate's role in emotion regulation and its clinical relevance to therapy and prognosis of mood disorders |
| Kong (2015a) | To explore the neural basis of SWB by correlating the regional fALFF with the self-reported SWB of healthy individuals. | Findings provide the first evidence that spontaneous brain activity in multiple regions associated with sensation, social perception, cognition, and emotion contributes to cognitive well-being, whereas the spontaneous brain activity in only one emotion-related region contributes to affective well-being, suggesting that the spontaneous activity of the human brain reflect the efficiency of SWB. |
| Kong (2015b) | To identify the brain regions underlying this construct by correlating individuals' psychological resilience scores with the regional homogeneity (ReHo) using resting-state fMRI and then examined how these resilience-related regions predicted life satisfaction in a sample of healthy young adults. | Spontaneous activity of the human brain reflects the efficiency of psychological resilience and highlight the right dACC within the salience network as a neural substrate linking psychological resilience and life satisfaction. |
| Kong (2015c) | To correlate individuals EWB scores with the regional fALFF of the brain, and then exam-ined how personality traits predicted EWB-related spontaneous brain activity. | This result provides the first evidence that EWB is associated with personality traits through different neural substrates. |
| Kong (2016) | To evaluate the regional homogeneity (ReHo) that measures the local synchronization of spontaneous brain activity in a large sample using resting-state fMRI. | Study provides the first evidence for linking individual differences in dispositional mindfulness to spontaneous brain activity and demonstrates that dispositional mindfulness engages multiple brain |

| | | |
|---|---|---|
| | | mechanisms that differentially influence hedonic and eudaimonic well-being. |
| Kong (2018) | To investigate the neural substrate for psychological resilience and the underlying mechanism for how psychological resilience enhances SWBin the healthy brain by employing fALFF measured with resting-state fMRI. | Provided further evidence for functional neural substrates of psychological resilience and reveals a potential mechanism that psychological resilience mediates the effect of spontaneous brain activity on SWB. |
| Kujawa (2015) | To evaluate prospective associations between the feedback negativity (FN) and positive emotionality (PE) from early to late childhood. | Results contributed to understanding the neural correlates of PE and suggest that the FN and PE may be related to the same biobehavioral approach system. |
| Kwon (2021) | To elucidate differences in the association of these needs and FC of reward processing and emotion regulation between individuals with high and low life satisfaction. | Individuals' perceived life satisfaction affects the relationship between the neural mechanism for reward processing and emotion regulation and basic psychological needs support. Psychological need satisfactions seem to have an emotional impact by acting as a contradictory brain mechanism between individuals with high and low life satisfaction. |
| Kyeong (2020) | To investigate the effect of two positive and negative self-talks on the functional connectome with respect to life satisfaction and its relationships with basic psychological needs. | Self-criticism produces more noticeable negative changes in the brain than the positive changes of self-respect. Individuals with low life satisfaction may be more vulnerable to be negatively affected not only by self-criticism but also self-respect than individuals with high life satisfaction. The satisfaction of basic psychological needs can play a mediating role in the effects of self-talk tasks differently concerning life satisfaction. |
| Larson (2010) | To investigate the relationship between measures of positive personality traits, including satisfaction with life, dispositional optimism, and PA, and indices of performance monitoring in a sample of 45 neurologically-healthy individuals. | Increased satisfaction with life was associated with decreased (i.e., less negative) error-related negativity (ERN) amplitude. Dispositional optimism and PA were not related to ERN amplitude. There were no relationships between measures of positive personality and the post-error positivity or behavioral indices. Findings are consistent with an affective salience interpretation of the ERN, with errors potentially being less meaningful for individuals with higher satisfaction with life. |
| Lewis (2014) | To examine whether regional GM volume was associated with eudaimonic well-being. | Eudaimonic well-being was positively associated with right insular cortex GM volume. This association was also reflected in three of the sub-scales of eudaimonia: personal growth, positive relations and purpose in life. Positive relations also showed a significant association with left insula volume. No other significant associations were observed, although personal growth was marginally associated with left insula, and purpose in life exhibited a marginally significant negative association with middle temporal gyrus GM volume. |
| Li (2011) | To investigate the neural mechanisms underlying vital loss decisions. | Vital loss decisions involve emotions and cannot be adequately captured by cold computation of minimizing losses. |
| Li (2016) | To decode emotional states and determine whether participants are able to modulate positive emotion networks by real-time fMRI neuro-feedback training using multivariate voxel pattern analysis-based online support vector machine. | Humans are able to voluntarily modulate positive emotion networks, leading to exciting applications in the treatment of various neurological and psychiatric disorders. |
| Lichev (2015) | To examine for the first time the relationship between trait emotional awareness and | People with high emotional awareness show stronger affective reactivity and more activation in brain areas involved in emotion processing and simulation during the perception of masked happy facial |

| | | |
|---|---|---|
| | automatic brain reactivity to positive and negative facial emotions in healthy adults. | expression than people with low emotional awareness. High emotional awareness appears to be characterized by an enhanced PA resonance to others at an automatic processing level. |
| Liu (2015) | To investigate how affective arousal regulates the reward-based choice behavior from the behavioral, model-fitting, and ERP perspectives using a mixed-design with both within- and between-subjects comparisons of the facial-expression primes in a probabilistic learning task. | Affective arousal negatively regulates reward-related choice, probably through overweighting with negative feedback. |
| Lorenzetti (2009) | To investigate pituitary gland volume (PGV) in currently depressed patients and individuals with a history of depression but who are currently in remission. | PGV does not appear to be a marker of current or past MDD in adult patients. |
| Love (2012) | To examine how oxytocin gene variation is associated with stress-induced dopaminergic neurotransmission in a healthy human sample. | Female rs4813625 C allele carriers demonstrated greater stress-induced dopamine release, measured as reductions in receptor availability from baseline to the pain-stress condition relative to female GG homozygotes. No significant differences were detected among males. Authors also observed that female rs4813625 C allele carriers exhibited higher attachment anxiety, higher trait anxiety and lower emotional well-being scores. In addition, greater stress-induced dopamine release was associated with lower emotional well-being scores in female rs4813625 C allele carriers. |
| Luo (2014) | To examine whether the local synchronization of spontaneous brain activities was associated with trait happiness. | The local synchronization of BOLD signal is altered in unhappy individuals. The regions implicated in this alteration partly overlapped with previously identified default mode network, emotional circuitry, and rewarding system, suggesting that these systems may be involved in happiness. |
| Luo (2017) | To investigate how the individual tendency to eudaimonic well-being relative to hedonic well-being, measured using eudaimonic and hedonic balance (EHB) index, is reflected in the functional and structural features of a key network of well-being. | The relative dominance of one form of well-being to the other is reflected in the morphometric characteristics and intrinsic functions of DMN. |
| Ma (2015) | To examine how a variant of the serotonin-transporter polymorphism (5-HTTLPR), which affects the expression and function of 5-HTT, influenced the acute effects of an SSRI (citalopram) on emotion-related brain activity in healthy adults. | 5-HTTLPR modulated the acute effects of citalopram on neural responses to negative emotions. Specifically, relative to placebo, citalopram increased amygdala and insula activity in l/l but not s/s homozygotes during perception of fearful faces. Similar analyses of brain activity in response to happy faces did not show any significant effects. Combined pharmacogenetic and functional imaging results provide a neurogenetic mechanism for discrepant acute effects of SSRIs. |
| Martikainen (2015) | To address the role of striatal dopamine (DA) D2/D3 receptor (D2/D3R) function in chronic non-neuropathic back pain (CNBP) by comparing CNBP patients and healthy controls using PET and the D2/D3R-selective radioligand raclopride. | CNBP is associated with adaptations in ventral striatal D2/D3Rfunction, which, together with endogenous opioid system function, contribute to the sensory and affective-motivational features of CNBP. |
| Martin-Soelch (2021) | Aims to test (1) whether increased family risk of depression is associated with blunted neural and self-reported reward responses. (2) the stress-reward interactions at the neural level. | Increased family risk of depression was associated with specific striatum reactivity to reward in a stress condition, and support previous findings that ventral striatal reward-related response is associated with PA. A new unexpected finding is the negative association between NA and reward-related ventral striatal activation in the control group (HC). |

| | | |
|---|---|---|
| Mathiak (2013) | Describes for the first time neural contributions to boredom, using a video game as complex virtual environment. | Confirmed that PA and NA are separable constructs, reflected by distinct neural patterns. PA may be associated with afferent limbic activity whereas NA with affective control. |
| Matsunaga (2016) | Investigate the interaction between the two aspects of happiness and investigate if it could be explained by the interaction between structure and function in certain brain regions. | Results provide convergent structural and functional evidence that the rACC is related to happiness and suggest that the interaction between structure and function in the rACC may explain the trait–state interaction in happiness. |
| McPhee (2021) | To investigate the neurocognitive effects of b*acopa monnieri* (BM) in a randomized, double-blind, placebo-controlled trial asked 28 healthy adults aged over 55 years to complete cognitive training (CT) 3 hours weekly for 12 weeks. | Given the exploratory outcomes and inconsistent findings between the behavioral and neuroimaging data, a larger study is needed to confirm the synaptogenic mechanisms of BM. |
| Memarian (2017) | To investigate whether the neural correlates of affect labeling predict improvements in measures of psychological and physical well-being in general and psychological and physical health benefits of expressive writing in particular. | This study demonstrates the substantial merit of supervised machine learning for real-world outcome prediction in social and affective neuroscience. |
| Mennella (2017) | To evaluate discrete changes in alpha power at left and right sites, as well as in PA and NA, anxiety and depression by employing a neuro-feedback training to increase frontal alpha asymmetry (right-left). | Findings provide a strong rationale for the use of frontal alpha asymmetry neurofeedback for the reduction of NA and anxiety in clinical settings. |
| Miskowiak (2008) | To explore the effects of Epo (40 000 IU) vs. saline on self-reported mood and on neural and cognitive function in healthy volunteers 3 days post-administration to test the reliability of the rapid mood improvement and its neuropsychological basis. | Characterization of the effects of Epo in a clinically depressed group is warranted. |
| Morelli (2018) | To test for responsiveness to different types of rewards by assessing individuals' neural sensitivity to personal vs. vicarious monetary reward outcomes and explored how responses to each related to prosociality and well-being. | Findings highlight the value of independently assessing responsiveness to different types of reward and illuminate affective mechanisms that may promote prosocial behavior and well-being. |
| Mrazek (2016) | To study how much the adult brain can be shaped by experience using an intensive multifaceted intervention. | Findings point to higher limits for rapid and concurrent cognitive, affective, and neural plasticity than is widely assumed. |
| Nardo (2011) | To investigate the distribution of the regional cerebral blood flow in occupational-related PTSD subjects and to seek possible correlations between brain perfusion and self-rating scales in order to cross-check their diagnostic value and to look for their neural correlates. | Findings support the involvement of insular, cingulate and parietal cortices (as well as the basal ganglia) in the pathogenesis of PTSD and in the processing of related SWB and distress. |
| Oetken (2017) | To investigate the interaction of mood and self-perception in 20 healthy participants using fMRI. | Findings in healthy research participants suggest a compensatory mechanism during sad mood induction to maintain a positive self-image, which is supported by activation of limbic and fronto-temporal cortex. Studies in clinically depressed populations could reveal whether this compensatory mechanism is aberrant. |
| Park (2017) | To investigate how generosity is linked to happiness on the neural level using fMRI. | Top–down control of striatal activity plays a fundamental role in linking commitment-induced generosity with happiness. |

| | | |
|---|---|---|
| Plitnick (2010) | To explore the roles that long- and short-wavelength lights have on momentary mood and alertness at night. | The red and the blue lights increased electroencephalographic beta power (12–30 Hz), reduced sleepiness, and increased PA relative to the previous dim-light period indicating that alertness and mood can be affected by light without necessarily stimulating the melatonin pathway. The impact of light was modest, however, compared to the increase in fatigue over the course of the night |
| Puccetti (2021) | Using representational similarity analysis to test whether individual differences in amygdala persistence following affective images was linked with daily affective experience and PWB. | Day-to-day experiences of PA comprise a promising intermediate step that links individual differences in neural dynamics to complex judgements of PWB. |
| Radsepehr (2019) | To investigate the effect of emotions (negative and positive affects) and gender on the activity of different brain regions and their relationship with personality traits of extraversion, introversion, neuroticism, and stability. | Regional brain activity is different under positive attitude and negative attitude inductions, but gender does not affect it. Also, personality traits are effective in the activity of some brain regions. |
| Rohr (2015) | First attempt to directly target the link between emotional traits and executive functions using resting-state fMRI analysis. | Individual differences in the ability to inhibit NA are mediated by prefrontal–limbic pathways, while the ability to be positive and use rewarding information is mediated by a network that includes the amygdala and thalamostriatal regions. |
| Sailer (2016) | To investigate which brain regions code hedonic experience during long-lasting pleasant touch. | Long-lasting stroking was processed in similar areas as shorter-lasting stroking. The decreased activation in somatosensory cortices over time may represent stimulus habituation, whereas increased activation in OFC and putamen may relate to the stimulation's subjective reward value. This involvement of reward-related brain circuits can facilitate maintenance of long-lasting social touch interactions. |
| Sanchez (2015) | Using fMRI to investigate whether different states of attention control would modulate amygdala responses to highly unpleasant pictures relative to neutral and whether this modulation would be influenced by the PA trait. | Subjects with high PA exhibit lower amygdala reactivity to distracting unpleasant pictures. In conclusion, the current study suggests that P PA modulates attention on unpleasant pictures, therefore attenuating emotional responses. |
| Sanger (2018) | To investigate the efficacy of a school-based mindfulness curriculum delivered by schoolteachers to older secondary school students (16–18 years) in a non-randomized controlled study. | In-school mindfulness training for adolescents has scope for increasing awareness of socially relevant emotional stimuli, irrespective of valence, and thus may decrease vulnerability to depression. |
| Sato (2005) | To investigate the effects of value (correct or incorrect) and reward magnitude (no, small or large) on feedback negativity and P300. | Value and reward magnitude are processed separately in the brain. |
| Sato (2015) | To investigate the neural substrates of subjective happiness using structural magnetic resonance imaging and questionnaires that assessed subjective happiness, the intensity of positive and negative emotional experiences, and purpose in life. | The precuneus mediates subjective happiness by integrating the emotional and cognitive components of happiness. |
| Sato (2019) | To investigate how the neural activity in the right precuneus, as well as the neural functional coupling between this and other regions, could be related to Subjective Happiness Scale scores. | Together with other evidence on the information-processing functions of these brain regions, findings suggest the possibility that subjective happiness is associated with a reduction in self-referential mental processes, which are well integrated with emotional processing. |

| | | |
|---|---|---|
| Schmitt (2019) | To employ fMRI to unravel associated neuronal changes of the emotional face-processing network in response to acute exercise. | This study provides first in vivo evidence that acute strenuous exercise interferes with emotional face-processing brain regions in an emotion type-specific manner. |
| Schneider (1997) | To replicate the previous $H_2{}^{15}$ O-PET study with high spatial resolution and fMRI and examine signal intensities as equivalents of regional cerebral blood flow during the experience of sadness and happiness. | Provides converging evidence that supports the potential of fMRI for advancing the understanding of neural substrates for emotional experience in humans. |
| Schöne (2018) | To study changes in behavioral performance of 34 participants during a multiple object tracking (MOT) task that taps core cognitive processes, namely sustained selective visual attention and spatial working memory. | This novel way of combining MOT and steady-state visually evoked potential provides the important insight that mindful breath awareness meditation may lead to refinements of attention networks, enabling more efficient use of attentional resources. |
| Shi (2016) | To investigate neural correlates of actual/ideal self-discrepancy and their associations with the serotonin transporter promoter polymorphism (5-HTTLPR) that moderates human affective states during self-reflection. | Revealed neural substrates of actual/ideal self-discrepancy and their associations with affective states that are sensitive to individuals' genetic makeup. |
| Shi (2019) | Using resting-state seed-based FC to explore the neural basis of psychological resilience and its association with PA in a big healthy sample. | Findings extend previous studies by revealing the FC basis of psychological resilience and highlighting the left OFC-IFG connectivity as a neural substrate linking PA and psychological resilience. |
| Singleton (2014) | To study the changes in brainstem GM concentration following a 8-week-mindfulness-based stress reduction (MBSR) course and the role of the pons and raphe in mood and arousal underlying changes in PWB. | This preliminary study suggests a neural correlate of enhanced PWB. The identified brain areas include the sites of synthesis and release of the neurotransmitters, norepinephrine and serotonin, which are involved in the modulation of arousal and mood, and have been related to a variety of affective functions as well as associated clinical dysfunctions. |
| Spironelli (2020) | To investigate frontal asymmetry in major depressive disorders (MDD), using language as a probe to test the integrity of large inter- and intra-hemispheric networks and processes, starts from an evolutionistic view of psychiatric disorders. | Language represents the human process that requires the largest level of integration between and within the hemispheres; thus, language asymmetry was a valid probe to test the left frontal alteration encompassing highly impairing psychiatric disorders, such as schizophrenia and MDD. |
| Suslow (2015) | To investigate the relationship of implicit (versus explicit) affectivity with the recognition of briefly presented affective body expressions. | Only implicit NA (but not explicit affect) was correlated with correct labeling performance for angry body posture. As expected, implicit NA associated with activation of the subcortical network in response to fearful and angry expression (compared to neutral expression). Responses of the caudate nucleus to affective body expression were especially associated with its recognition. It appears that processes of rapid recognition of affects from body postures could be facilitated by an individual’s implicit NA. |
| Talmon (2021) | To study the extent to which there is neurocognitive heterogeneity in social anxiety disorder (SAD) in two independent samples of patients through a self-referential encoding task and self-reports of childhood maltreatment, SWB, and emotion regulation using fMRI. | These findings reveal neurocognitive heterogeneity in SAD and its relationship to emotional maltreatment. |

| | | |
|---|---|---|
| Urry (2004) | To explore the neural correlates of eudaimonic well-being, hedonic well-being, and positive affect using resting EEG. | Greater left than right superior frontal activation was associated with higher levels of both forms of well-being. Hemisphere-specific analyses documented the importance of goal-directed approach tendencies beyond those captured by approach-related PA for eudaimonic but not for hedonic well-being. Appropriately engaging sources of appetitive motivation, characteristic of higher left than right baseline levels of pre- frontal activation, may encourage the experience of well-being. |
| Van Reekum (2007) | Using fMRI to examine whether individual differences in amygdala activation in response to negative relative to neutral information are related to differences in the speed with which such information is evaluated, the extent to which such differences are associated with mPFC function, and their relationship with measures of trait anxiety and PWB. | People high in PWB effectively recruit the ventral ACC when confronted with potentially aversive stimuli, manifest reduced activity in subcortical regions such as the amygdala, and appraise such information as less salient as reflected in slower evaluative speed. |
| Vanderhasselt (2013) | To investigate the effects on cognitive control considering negative versus positive material | After tDCS over the left dlPFC (and not sham control stimulation), authors observed more negative N450 amplitudes along with faster reaction times when inhibiting a habitual response to happy compared to sad facial expressions. Gender did not influence the effects of tDCS on cognitive control for emotional information. In line with the Valence Theory of side-lateralized activity, this stimulation protocol might have led to a left dominant (relative to right) prefrontal cortical activity, resulting in augmented cognitive control specifically for positive relative to negative stimuli. |
| Velikova (2017) | To test the hypothesis that training can affect cognitive functions by repeating and expanding the previous study that demonstrated that a 12-week lasting self-guided positive imagery training had a positive effect on the psycho-emotional state of healthy subjects and was associated with an increase in FC in the brain. | Comparison of EEG post-training vs. pre-training demonstrated a significant reduction in current source density (CSD) after the training in the left hemisphere (insular cortex, frontal and temporal lobes in delta, theta and alpha1 bands). The observed changes were presented only in the subgroup with initial subthreshold depressive symptomatology. A negative correlation was found between perception of emotions test and CDS in the left insular cortex for theta band. No significant differences were observed when data from EEG and cognitive tests obtained during pre-training were compared with baseline values. |
| Volkow (2011) | To test whether the OFC and the cingulate underlie positive emotionality (PEM) by accessing the correlation between baseline brain glucose metabolism (measured with PET and [18F] fluorodeoxyglucose) and scores on PEM measurement. | Results corroborate the involvement of orbitofrontal and cingulate regions in PEM, which is considered a trait that protects against substance use disorders. As dysfunction of OFC and cingulate is a hallmark of addiction, these findings support a common neural basis underlying protective personality factors and brain dysfunction underlying substance use disorders. Authos also uncovered an association between PEM and baseline metabolism in regions from the DMN, which suggests that PEM may relate to global cortical processes that are active during resting conditions (introspection, mind wandering). |
| Wang (2020) | To investigate the dynamic characteristic with high temporal precision in the default network (DN) after sleep deprivation using resting-state EEG. | The results reveal the strong relationship between the uncoupling of DN and the mood state of feeling down. This research may contribute towards a better understanding of the mood and cognition processing after sleep loss. |
| Waytz (2015) | Numerous lines of research also suggest that the tendency for mental simulation is associated with enhanced meaning. The present research tests this association specifically examining the relationship between two forms of simulation (temporal and spatial) and meaning in life. | This research sheds light on an important determinant of meaning in life and suggests that undirected mental simulation benefits PWB. |

| | | |
|---|---|---|
| Woods (2020) | To examine associations between neural response to this task and positive friendship affect and behavior (e.g., shared PA between friends in the laboratory and in daily life) and evaluate the association between adolescent neural, behavioral, and self-reported emotional responses to positive affect PA displayed by a close friend. | Provided information on the role of lateral prefrontal cortex and anterior insula in enjoyment of friendships during adolescence. |
| Xu (2018) | Using EEG to identify whether the intentional increase of SWB through positive psychological intervention (PPIs) was associated with greater tonic left frontal activation. | Adaptive emotion regulation, which is characteristic of greater tonic left frontal activation, reflects the efficiency of PPIs and highlights the frontal alpha EEG asymmetry as a neural substrate linking PPIs and mental health. |
| Yu (2012) | Using the emotion-priming paradigm to examine the neural mechanisms underlying the relationship between SWB and the processing of emotional stimuli by recording ERP relevant to emotion probe words. | Provided electrophysiological evidence for the relationship between SWB and emotion processing. |
| Zhang (2014) | To estimate the effects of microgravity on the central nervous activity and its underlying influencing mechanisms. | The results obtained in the study support the use of an approach which combines a multi-factor influential mechanism hypothesis. The changes in the EEG data may be influenced by both cardiovascular and cognitive effects. |
| Zhang (2015) | To study which nodes are critical for affective information flow, which hubs of affective functional networks are better structured for local versus global processes, or whether the graph-theoretic characteristics of these hubs correlate with the PANAS scores. | Provided important evidence for the organizational principles of the human brain functional connectome during the processing of affective information. |
| Zhang (2017) | Using the ERP version of the Cyberball paradigm to investigate the emotions and neural activities in depressive patients during social inclusion and exclusion simultaneously to explore neuropsychological mechanisms. | The behavioral and electrophysiological results indicated that the interpersonal problems in depressive patients were mainly due to deficits in processing the pleasurable social stimuli rather than aversive social cues. |
| Zubieta (2003) | To examine the involvement of μ-opioid neurotransmission in the regulation of affective states in healthy human volunteers. | These data demonstrate dynamic changes in μ-opioid neurotransmission in response to an experimentally induced NA state. The direction and localization of these responses confirms the role of the μ-opioid receptor system in the physiological regulation of affective experiences in humans. |

***Notes.*** dACC = dorsal anterior cingulate cortex; dlPFC = dorsolateral prefrontal cortex; EEG = electroencephalography; ERP = event-related potential; fALFF = fractional amplitude of low-frequency fluctuations; FC = functional connectivity; fMRI = functional magnetic resonance imaging; GM = Gray matter; MDD = major depressive disorders; mPFC = medial prefrontal cortex; NA = Negative affect; OFC = orbitofrontal cortex; PA = Positive affect; PANAS = Positive and Negative Affect Schedule/Scale; PET = positron emission tomography; PWB = psychological well-being; PTSD = post-traumatic stress disorder; sLORETA = standardized low-resolution brain electromagnetic tomography; SWB = subjective well-being; tDCS = Transcranial Direct-Current Stimulation; TMS = Transcranial Magnetic Stimulation.

**Table S4**

*Journal Published (N=95)*

| Journal | # of papers | JCR indicator |
|---|---|---|
| *NeuroImage* | 9 | 8.011 |
| *Social Cognitive and Affective Neuroscience* | 9 | 5.199 |
| *Frontiers in Human Neuroscience* | 6 | 4.111 |
| *American Journal of Psychiatry* | 4 | 19.590 |
| *Frontiers in Psychology* | 4 | 4.426 |
| *Cognitive Affective & Behavioral Neuroscience* | 3 | 3.790 |
| *PLoS ONE* | 3 | 4.069 |
| *Scientific Reports* | 3 | 5.516 |
| *Biological Psychiatry* | 2 | 14.454 |
| *Biological Psychology* | 2 | 3.965 |
| *Frontiers in Psychiatry* | 2 | 5.556 |
| *Journal of Neuroscience* | 2 | 7.455 |
| *Neuropsychologia* | 2 | 3.563 |
| *Neuroscience Letters* | 2 | 2.892 |
| *Psychiatry Research - Neuroimaging* | 2 | 2.831 |
| *Psychological Medicine* | 2 | 9.658 |
| *Psychological Science* | 2 | 9.647 |
| *Social Neuroscience* | 2 | 2.826 |
| Other journals | 34 | — |

*Notes.* JCR = 5-year JCR impact factor.

**Table S5**

*Times EWB questionnaires and constructs were used and investigated across the N=95 studies*

| EWB Questionnaires | EWB Constructs | | | | | Times EWB questionnaire used |
|---|---|---|---|---|---|---|
| | Goal pursuit | Life Satisfaction | Positive affect | Quality of life | Sense of meaning | |
| Affect and Arousal Scale (AFARS) | | | ✓ | | | 1 |
| Life Orientation Test-Revised (LOT-R) | | ✓ | | | | 2 |
| Meaning in Life Questionnaire (MLQ) | ✓ | ✓ | | | ✓ | 1 |
| Multidimensional Personality Questionnaire Well-being Scale (MPQ-WB) | | ✓ | ✓ | | | 1 |
| Positive and Negative Affect Schedule (PANAS) | | ✓ | ✓ | | | 65 |
| Positive and Negative Affect Schedule for Children (PANAS-C) | | ✓ | ✓ | | | 2 |
| Quality of Life scale (CASP-19) | | ✓ | | ✓ | | 1 |
| Ryff's Scales of Psychological Well-Being (RPWB) | ✓ | ✓ | ✓ | | ✓ | 11 |
| Satisfaction with Life Scale (SWLS) | | ✓ | | | | 17 |
| Scale of Happiness of the Memorial University of Newfoundland (MUNSH) | | ✓ | ✓ | | | 1 |
| Subjective Happiness Scale (SHS) | | ✓ | ✓ | | | 10 |
| World Health Organization Quality-of-Life Brief Scale (WHOQOL-Bref) | | ✓ | | ✓ | ✓ | 1 |
| World Health Organization Well-Being Index (WHO) | ✓ | ✓ | ✓ | | ✓ | 2 |
| Frequency of EWB construct being investigated out of 95 studies | 13 | 94 | 86 | 2 | 14 | — |

*Notes.* Each study includes one or more than one EWB questionnaire(s) and each questionnaire investigates one or more than one EWB construct(s).

**Figure S1**

*A World Map with Table Showing the Percentage and Number of Included Studies Grouped by Continent*

| Continent | World Population Share | Studies (n=95) |
|---|---|---|
| Asia | 59.54% | 33.8% (n=32) |
| Africa | 17.20% | 0.00% (n=0) |
| Europe | 9.59% | 26.4% (n=25) |
| North America | 7.60% | 30.5% (n=29) |
| South America | 5.53% | 2.1% (n=2) |
| Australia/ Oceania | 0.55% | 6.3% (n=6) |
| Antarctica | 0.00% | 0.00% (n=0) |
| Not reported | — | 1.05% (n=1) |

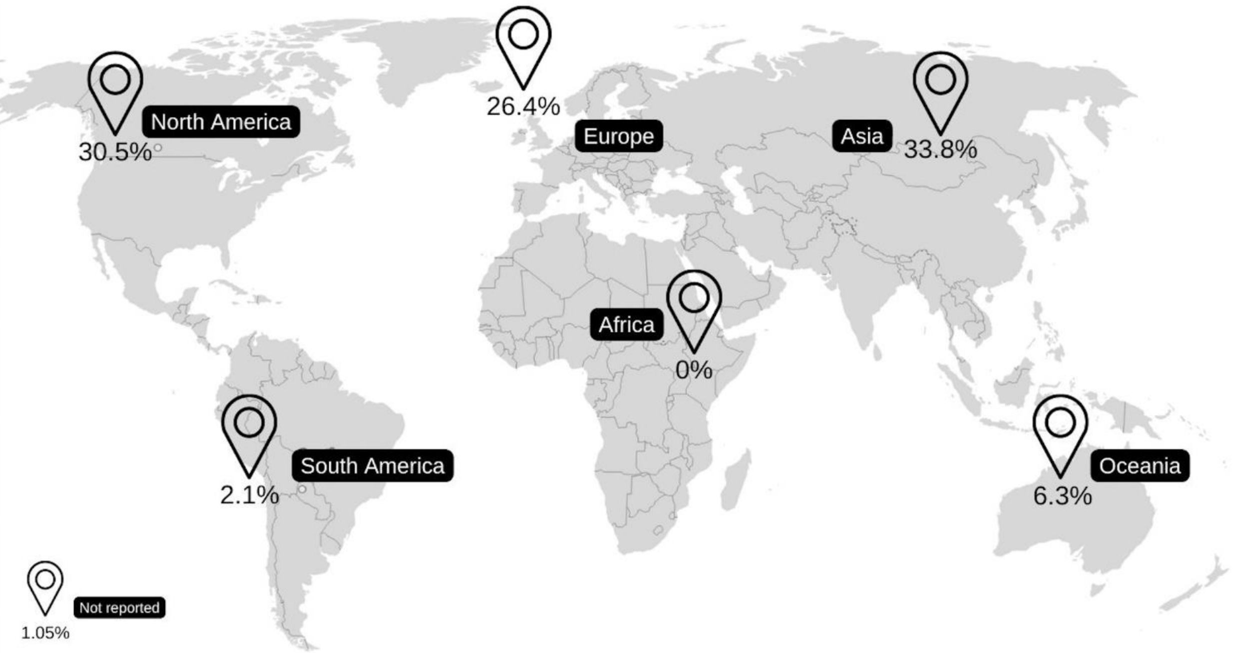


*Notes.* Information about 94 studies was available, since one study did not report the country of the study. World population share data obtained from The World Bank, 2021.
