# Supplementary material for: Brain imaging studies of emotional well-being: a scoping review
Source: Front Psychol. 2024 Jan 5;14:1328523. doi: 10.3389/fpsyg.2023.1328523 (PMC10799564; doi:10.3389/fpsyg.2023.1328523)
Supplement: Supplementary file 2 [file Data_Sheet_1.pdf]

**PubMed**

emotional well-being [tiab] OR emotional wellbeing [tiab] OR psychological well-being [tiab] OR psychological wellbeing [tiab] OR subjective well-being [tiab] OR subjective wellbeing [tiab] OR life satisfaction [tiab] OR happiness [tiab] OR happy [tiab] OR positive emotion* [tiab] OR flourish* [tiab] OR Eudaimoni* [tiab] OR evaluative well-being [tiab] OR evaluative wellbeing [tiab] OR hedonic well-being [tiab] OR hedonic wellbeing [tiab] OR experiential well-being [tiab] OR experiential wellbeing [tiab] OR spiritual well-being [tiab] OR spiritual wellbeing [tiab] OR positive affect [tiab] OR meaning in life [tiab]

AND

magnetic resonance imag* [tiab] OR functional MRI [tiab] OR electroencephalogra* OR event related* [tiab] OR event-related* [tiab] OR magnetic resonance spectroscop* [tiab] OR positron emission [tiab] OR single-photon emission [tiab] OR magnetoencephalogra* [tiab] OR Transcranial magnetic stimulation [tiab] OR Transcranial direct current stimulation [tiab] OR diffusion weighted [tiab] OR diffusion-weighted [tiab] OR diffusion tensor [tiab] OR diffusion-tensor [tiab] OR diffusion MRI [tiab] OR diffusion imaging [tiab] OR MRI [tiab] OR fMRI [tiab] OR EEG [tiab] OR ERP [tiab] OR MRS [tiab] OR PET [tiab] OR SPECT [tiab] OR MEG [tiab] OR TMS [tiab] OR tDCS [tiab] OR DWI [tiab] OR DTI [tiab]

AND

brain* [tiab] OR neur* [tiab]

Saturday, July 10, 2021 1:33:53 AM

| # | Query | Limiters/Expanders | Last Run Via | Results |
|---|---|---|---|---|
| S4 | (TI brain* OR neur* OR AB brain* OR neur* OR SU brain* OR neur* OR KW brain* OR neur*) AND (S1 AND S2 AND S3) | Expanders - Apply equivalent subjects Search modes - Boolean/Phrase | Interface - EBSCOhost Research Databases Search Screen - Advanced Search Database - APA PsycInfo | 2,244 |
| S3 | TI ( brain* OR neur* ) OR AB ( brain* OR neur* ) OR SU ( brain* OR neur* ) OR KW ( brain* OR neur* ) | Expanders - Apply equivalent subjects Search modes - Boolean/Phrase | Interface - EBSCOhost Research Databases Search Screen - Advanced Search Database - APA PsycInfo | 862,807 |
| S2 | TI ( "magnetic resonance imag*" OR "functional MRI" OR electroencephalogra* OR “event related*” OR event-related* OR "magnetic resonance spectroscop*" OR "positron emission" OR "single-photon emission" OR magnetoencephalogra* OR "Transcranial magnetic stimulation" OR "Transcranial direct current stimulation" OR "diffusion weighted” OR “diffusion-weighted” OR "diffusion tensor" OR “diffusion-tensor” OR “diffusion MRI” OR “diffusion imaging” OR MRI OR fMRI OR EEG OR ERP OR MRS OR | Expanders - Apply equivalent subjects Search modes - Boolean/Phrase | Interface - EBSCOhost Research Databases Search Screen - Advanced Search Database - APA PsycInfo | 209,384 |

PET OR SPECT OR
MEG OR TMS OR tDCS
OR DWI OR DTI ) OR AB
( "magnetic resonance
imag*" OR "functional
MRI" OR
electroencephalogra* OR
“event related*” OR
event-related* OR
"magnetic resonance
spectroscop*" OR
"positron emission" OR
"single-photon emission"
OR
magnetoencephalogra*
OR "Transcranial
magnetic stimulation" OR
"Transcranial direct
current stimulation" OR
"diffusion weighted” OR
“diffusion-weighted” OR
"diffusion tensor" OR
“diffusion-tensor” OR
“diffusion MRI” OR
“diffusion imaging” OR
MRI OR fMRI OR EEG
OR ERP OR MRS OR
PET OR SPECT OR
MEG OR TMS OR tDCS
OR DWI OR DTI ) OR SU
( "magnetic resonance
imag*" OR "functional
MRI" OR
electroencephalogra* OR
“event related*” OR
event-related* OR
"magnetic resonance
spectroscop*" OR
"positron emission" OR
"single-photon emission"
OR
magnetoencephalogra*
OR "Transcranial

magnetic stimulation" OR "Transcranial direct current stimulation" OR "diffusion weighted” OR “diffusion-weighted” OR "diffusion tensor" OR “diffusion-tensor” OR “diffusion MRI” OR “diffusion imaging” OR MRI OR fMRI OR EEG OR ERP OR MRS OR PET OR SPECT OR MEG OR TMS OR tDCS OR DWI OR DTI ) OR KW ( "magnetic resonance imag*" OR "functional MRI" OR electroencephalogra* OR “event related*” OR event-related* OR "magnetic resonance spectroscop*" OR "positron emission" OR "single-photon emission" OR magnetoencephalogra* OR "Transcranial magnetic stimulation" OR "Transcranial direct current stimulation" OR "diffusion weighted” OR “diffusion-weighted” OR "diffusion tensor" OR “diffusion-tensor” OR “diffusion MRI” OR “diffusion imaging” OR MRI OR fMRI OR EEG OR ERP OR MRS OR PET OR SPECT OR MEG OR TMS OR tDCS OR DWI OR DTI )

| | | | | |
|---|---|---|---|---|
| S1 | TI ( "emotional well-being" OR "emotional wellbeing" OR "psychological well-being" OR "psychological wellbeing" OR "subjective well-being" OR "subjective wellbeing" OR "life satisfaction" OR happiness OR happy OR "positive emotion*" OR flourish* OR Eudaimoni* OR "evaluative well-being" OR "evaluative wellbeing" OR "hedonic well-being" OR "hedonic wellbeing" OR "experiential well-being" OR "experiential wellbeing" OR "spiritual well-being" OR "spiritual wellbeing" OR "positive affect" OR "meaning in life" ) OR AB ( "emotional well-being" OR "emotional wellbeing" OR "psychological well-being" OR "psychological wellbeing" OR "subjective well-being" OR "subjective wellbeing" OR "life satisfaction" OR happiness OR happy OR "positive emotion*" OR flourish* OR Eudaimoni* OR "evaluative well-being" OR "evaluative wellbeing" OR "hedonic well-being" OR "hedonic wellbeing" OR "experiential well-being" OR "experiential wellbeing" OR "spiritual | Expanders - Apply equivalent subjects<br>Search modes - Boolean/Phrase | Interface - EBSCOhost Research Databases<br>Search Screen - Advanced Search<br>Database - APA PsycInfo | 91,328 |

well-being" OR "spiritual wellbeing" OR "positive affect" OR "meaning in life" ) OR SU ( "emotional well-being" OR "emotional wellbeing" OR "psychological well-being" OR "psychological wellbeing" OR "subjective well-being" OR "subjective wellbeing" OR "life satisfaction" OR happiness OR happy OR "positive emotion*" OR flourish* OR Eudaimoni* OR "evaluative well-being" OR "evaluative wellbeing" OR "hedonic well-being" OR "hedonic wellbeing" OR "experiential well-being" OR "experiential wellbeing" OR "spiritual well-being" OR "spiritual wellbeing" OR "positive affect" OR "meaning in life" ) OR KW ( "emotional well-being" OR "emotional wellbeing" OR "psychological well-being" OR "psychological wellbeing" OR "subjective well-being" OR "subjective wellbeing" OR "life satisfaction" OR happiness OR happy OR "positive emotion*" OR flourish* OR Eudaimoni* OR "evaluative well-being" OR "evaluative wellbeing" OR "hedonic well-being" OR "hedonic wellbeing" OR

"experiential well-being" OR "experiential wellbeing" OR "spiritual well-being" OR "spiritual wellbeing" OR "positive affect" OR "meaning in life" )

UNIVERSITY LIBRARIES

Skip navigation menu English Products

Web of Science™ Search Marked List 50 History Alerts Sign In Register

< BACK TO BASIC SEARCHES

# Advanced Search Query Builder

Search in: **All Databases** Collections: **All**

Add terms to the query search preview

Topic

Example: oil spill* mediterranean

And

Add to query

Less options

Exact search

Query Preview

((#1) AND #2) AND #3

+ Add date range Field Tags × Clear Search

Query #1

**(TI=("emotional well-being" OR "emotional wellbeing" OR "psychological well-being" OR "psychological wellbeing" OR "subjective well-being" OR "subjective wellbeing" OR "life satisfaction" OR happiness OR happy OR "positive emotion*" OR flourish* OR Eudaimoni* OR "evaluative well-being" OR "evaluative wellbeing" OR "hedonic well-being" OR "hedonic wellbeing" OR "experiential well-being" OR "experiential wellbeing" OR "spiritual well-being" OR "spiritual wellbeing" OR "positive affect" OR "meaning in life")) OR AB=("emotional well-being" OR "emotional wellbeing" OR "psychological well-being" OR "psychological wellbeing" OR "subjective well-being" OR "subjective wellbeing" OR "life satisfaction" OR happiness OR happy OR "positive emotion*" OR flourish***

**OR Eudaimoni* OR "evaluative well-being" OR "evaluative wellbeing" OR "hedonic well-being" OR "hedonic wellbeing" OR "experiential well-being" OR "experiential wellbeing" OR "spiritual well-being" OR "spiritual wellbeing" OR "positive affect" OR "meaning in life")**

Edit

Query #2

**(TI=("magnetic resonance imag*" OR "functional MRI" OR electroencephalogra* OR “event related*” OR event-related* OR "magnetic resonance spectroscop*" OR "positron emission" OR "single-photon emission" OR magnetoencephalogra* OR "Transcranial magnetic stimulation" OR "Transcranial direct current stimulation" OR "diffusion weighted” OR “diffusion-weighted” OR "diffusion tensor" OR “diffusion-tensor” OR “diffusion MRI” OR “diffusion imaging” OR MRI OR fMRI OR EEG OR ERP OR MRS OR PET OR SPECT OR MEG OR TMS OR tDCS OR DWI OR DTI)) OR AB=("magnetic resonance imag*" OR "functional MRI" OR electroencephalogra* OR “event related*” OR event-related* OR "magnetic resonance spectroscop*" OR "positron emission" OR "single-photon emission" OR magnetoencephalogra* OR "Transcranial magnetic stimulation" OR "Transcranial direct current stimulation" OR "diffusion weighted” OR “diffusion-weighted” OR "diffusion tensor" OR “diffusion-tensor” OR “diffusion MRI” OR “diffusion imaging” OR MRI OR fMRI OR EEG OR ERP OR MRS OR PET OR SPECT OR MEG OR TMS OR tDCS OR DWI OR DTI)**

Edit

Query #3

**(TI=(brain* OR neur*)) OR AB=(brain* OR neur*)**

Edit

History

| | | | | |
|---|---|---|---|---|
| 4 | ((#1) AND #2) AND #3 | Edit | Add to Search | 2,424 |
| 3 | (TI=(brain* OR neur*)) OR AB=(brain* OR neur*) | Edit | Add to Search | 4,222,067 |

| | | | | |
|---|---|---|---|---|
| 2 | (TI=("magnetic resonance imag*" OR "functional MRI" OR electroencephalogra* OR "event related*" OR event-related* OR "magnetic resonance spectroscop*" | Edit | Add to Search | 1,151,472 |
| 1 | (TI=("emotional well-being" OR "emotional wellbeing" OR "psychological well-being" OR "psychological wellbeing" OR "subjective well-being" OR "subjective | Edit | Add to Search | 153,737 |

© 2021 Clarivate
Training Portal
Product Support
Data Correction
Privacy Statement
Newsletter
Copyright Notice
Cookie Policy
Terms of Use
Follow Us

EBSCOhost

Saturday, July 10, 2021 1:44:09 AM

| # | Query | Limiters/Expanders | Last Run Via | Results |
|---|---|---|---|---|
| S4 | (TI brain* OR neur* OR AB brain* OR neur* OR SU brain* OR neur* OR KW brain* OR neur*) AND (S1 AND S2 AND S3) | Expanders - Apply equivalent subjects<br>Search modes - Boolean/Phrase | Interface - EBSCOhost Research Databases<br>Search Screen - Advanced Search<br>Database - ERIC | 64 |
| S3 | TI ( brain* OR neur* ) OR AB ( brain* OR neur* ) OR SU ( brain* OR neur* ) OR KW ( brain* OR neur* ) | Expanders - Apply equivalent subjects<br>Search modes - Boolean/Phrase | Interface - EBSCOhost Research Databases<br>Search Screen - Advanced Search<br>Database - ERIC | 27,797 |
| S2 | TI ( "magnetic resonance imag*" OR "functional MRI" OR electroencephalogra* OR “event related*” OR event-related* OR "magnetic resonance spectroscop*" OR "positron emission" OR "single-photon emission" OR magnetoencephalogra* OR "Transcranial magnetic stimulation" OR "Transcranial direct current stimulation" OR "diffusion weighted” OR “diffusion-weighted” OR "diffusion tensor" OR “diffusion-tensor” OR “diffusion MRI” OR “diffusion imaging” OR MRI OR fMRI OR EEG OR ERP OR MRS OR | Expanders - Apply equivalent subjects<br>Search modes - Boolean/Phrase | Interface - EBSCOhost Research Databases<br>Search Screen - Advanced Search<br>Database - ERIC | 5,375 |

PET OR SPECT OR MEG OR TMS OR tDCS OR DWI OR DTI ) OR AB ( "magnetic resonance imag*" OR "functional MRI" OR electroencephalogra* OR “event related*” OR event-related* OR "magnetic resonance spectroscop*" OR "positron emission" OR "single-photon emission" OR magnetoencephalogra* OR "Transcranial magnetic stimulation" OR "Transcranial direct current stimulation" OR "diffusion weighted” OR “diffusion-weighted” OR "diffusion tensor" OR “diffusion-tensor” OR “diffusion MRI” OR “diffusion imaging” OR MRI OR fMRI OR EEG OR ERP OR MRS OR PET OR SPECT OR MEG OR TMS OR tDCS OR DWI OR DTI ) OR SU ( "magnetic resonance imag*" OR "functional MRI" OR electroencephalogra* OR “event related*” OR event-related* OR "magnetic resonance spectroscop*" OR "positron emission" OR "single-photon emission" OR magnetoencephalogra* OR "Transcranial

magnetic stimulation" OR
"Transcranial direct
current stimulation" OR
"diffusion weighted” OR
“diffusion-weighted” OR
"diffusion tensor" OR
“diffusion-tensor” OR
“diffusion MRI” OR
“diffusion imaging” OR
MRI OR fMRI OR EEG
OR ERP OR MRS OR
PET OR SPECT OR
MEG OR TMS OR tDCS
OR DWI OR DTI ) OR
KW ( "magnetic
resonance imag*" OR
"functional MRI" OR
electroencephalogra* OR
“event related*” OR
event-related* OR
"magnetic resonance
spectroscop*" OR
"positron emission" OR
"single-photon emission"
OR
magnetoencephalogra*
OR "Transcranial
magnetic stimulation" OR
"Transcranial direct
current stimulation" OR
"diffusion weighted” OR
“diffusion-weighted” OR
"diffusion tensor" OR
“diffusion-tensor” OR
“diffusion MRI” OR
“diffusion imaging” OR
MRI OR fMRI OR EEG
OR ERP OR MRS OR
PET OR SPECT OR
MEG OR TMS OR tDCS
OR DWI OR DTI )

| | | | | |
|---|---|---|---|---|
| S1 | TI ( "emotional well-being" OR "emotional wellbeing" OR "psychological well-being" OR "psychological wellbeing" OR "subjective well-being" OR "subjective wellbeing" OR "life satisfaction" OR happiness OR happy OR "positive emotion*" OR flourish* OR Eudaimoni* OR "evaluative well-being" OR "evaluative wellbeing" OR "hedonic well-being" OR "hedonic wellbeing" OR "experiential well-being" OR "experiential wellbeing" OR "spiritual well-being" OR "spiritual wellbeing" OR "positive affect" OR "meaning in life" ) OR AB ( "emotional well-being" OR "emotional wellbeing" OR "psychological well-being" OR "psychological wellbeing" OR "subjective well-being" OR "subjective wellbeing" OR "life satisfaction" OR happiness OR happy OR "positive emotion*" OR flourish* OR Eudaimoni* OR "evaluative well-being" OR "evaluative wellbeing" OR "hedonic well-being" OR "hedonic wellbeing" OR "experiential well-being" OR "experiential wellbeing" OR "spiritual | Expanders - Apply equivalent subjects<br>Search modes - Boolean/Phrase | Interface - EBSCOhost Research Databases<br>Search Screen - Advanced Search<br>Database - ERIC | 12,683 |

well-being" OR "spiritual wellbeing" OR "positive affect" OR "meaning in life" ) OR SU ( "emotional well-being" OR "emotional wellbeing" OR "psychological well-being" OR "psychological wellbeing" OR "subjective well-being" OR "subjective wellbeing" OR "life satisfaction" OR happiness OR happy OR "positive emotion*" OR flourish* OR Eudaimoni* OR "evaluative well-being" OR "evaluative wellbeing" OR "hedonic well-being" OR "hedonic wellbeing" OR "experiential well-being" OR "experiential wellbeing" OR "spiritual well-being" OR "spiritual wellbeing" OR "positive affect" OR "meaning in life" ) OR KW ( "emotional well-being" OR "emotional wellbeing" OR "psychological well-being" OR "psychological wellbeing" OR "subjective well-being" OR "subjective wellbeing" OR "life satisfaction" OR happiness OR happy OR "positive emotion*" OR flourish* OR Eudaimoni* OR "evaluative well-being" OR "evaluative wellbeing" OR "hedonic well-being" OR "hedonic wellbeing" OR

"experiential well-being" OR "experiential wellbeing" OR "spiritual well-being" OR "spiritual wellbeing" OR "positive affect" OR "meaning in life" )
