# Supplementary material for: Brain imaging studies of emotional well-being: a scoping review
Source: Front Psychol. 2024 Jan 5;14:1328523. doi: 10.3389/fpsyg.2023.1328523 (PMC10799564; doi:10.3389/fpsyg.2023.1328523)
Supplement: Supplementary file 3 [file Data_Sheet_2.pdf]

Systematic Review - U24 EWB Imaging Subgroup

Abstract/title screening questions

<u>Title or Abstract Screening</u>

1. Does the title or abstract use English?

    a. Yes: continue screening

    b. No: stop screening

2. Does the title or abstract indicate that this is NOT a review/systematic review/meta-analysis/chapter?

    a. Yes: continue screening

    b. No: stop screening

3. Does the title or abstract indicate that this is NOT an animal study?

    a. Yes or Unsure/Unclear: continue screening

    b. No: stop screening

4. Does the abstract indicate that the study uses a quantitative design?

    a. Yes or Unsure/Unclear: continue screening

        -For example: regression, covariate, modeling, structural equation modeling, mean, standard deviation, correlation, variance, case-control study (a group with and without a disease)

    b. No: stop screening

        -For example: qualitative only: ethnography, action research, social observation, focus groups, case study research (usually one data point); case report

        - words that usually indicate reviews: overview, state of the science

5. Does the title or abstract indicate that a measure of emotional well-being (or its components) was used?

a. Yes: continue screening

- Key words (at least one of these terms MUST be included in the abstract/title): emotional well-being OR emotional wellbeing OR psychological well-being OR psychological wellbeing OR subjective well-being OR subjective wellbeing OR life satisfaction OR happiness OR happy OR positive emotion* OR flourish* OR Eudaimoni* OR evaluative well-being OR evaluative wellbeing OR hedonic well-being OR hedonic wellbeing OR experiential well-being OR experiential wellbeing OR spiritual well-being OR spiritual wellbeing OR positive affect OR meaning in life
- If someone manipulates happiness/positive emotion and measures some neural/physio response --We will INCLUDE these
- What if they measure trait level happiness and link that to a physio/neural response? --We will INCLUDE these
- Studies measuring participants' emotion recognition, identification, or perception (faces or intonation) -- we will EXCLUDE these
- Study includes a task in which participants are passive viewing faces (happy or positive emotions) -- we would EXCLUDE these.
- Study includes a measure of emotion regulation -- we would EXCLUDE these.
- Make sure emotional well-being is being MEASURED

b. No: stop screening

6. Does the title or abstract indicate that an imaging was used?

a. Yes: continue screening

-Key words (at least one of these terms MUST be included in the abstract/title): "magnetic resonance imag*" OR "functional MRI" OR electroencephalogra* OR "event related*" OR event-related* OR "magnetic resonance spectroscop*" OR "positron emission" OR "single-photon emission" OR magnetoencephalogra* OR "Transcranial magnetic stimulation" OR "Transcranial direct current stimulation" OR "diffusion weighted" OR "diffusion-weighted" OR "diffusion tensor" OR "diffusion-tensor" OR "diffusion MRI" OR "diffusion imaging" OR MRI OR fMRI OR EEG OR ERP OR MRS OR PET OR SPECT OR MEG OR TMS OR tDCS OR DWI OR DTI

- Notes: include studies conducted with neuro imaging via voxel-based morphometry. It is a method based on fMRI.

b. No: stop screening

7. Does the title or abstract indicate that an imaging was used in the brain?

a. Yes: continue screening

- Terms: brain* OR neur*
- It is clear that the imaging is from the brain (e.g., head). If it is unclear (maybe) - keep the paper

b. No: stop screening

- No: only if it is definitely (clear) that imaging was not from the brain/head (e.g., foot, hips, etc.)

8. Does the title or abstract indicate that a questionnaire was used to measure EWB?

- Terms: ("measure*" OR "assessment*" OR "self-report" OR "selfreport" OR "rating*" OR "scale*" OR "questionnaire" OR "Survey" OR "Instrument")

- a. Yes: continue screening (measure is included in the "Compiled measures list"
- b. No: stop screening
    - No: if includes a questionnaire that is not in the "Compiled measures list".

Decision: **Should this article be included?**

a. **Yes**, all 8 screening questions answered Yes or Unclear

b. **No**, at least one answer is definitely "No"
